# Mapping Royal Jelly’s Bioactive Profile by Liquid Chromatography Coupled to Trapped Ion Mobility Spectrometry and High-Resolution Mass Spectrometry

**DOI:** 10.1021/acs.jafc.5c04904

**Published:** 2025-09-08

**Authors:** Eleni S. Nastou, Dafni A. Preza-Mayo-Kataki, Panagiotis-Loukas P. Gialouris, Niki C. Maragou, Evagelos E. Gikas, Nikolaos S. Thomaidis

**Affiliations:** † Laboratory of Analytical Chemistry, Department of Chemistry, 68993 National and Kapodistrian University of Athens, Panepistimioupolis Zografou, Athens 15771, Greece; ‡ Department of Wine, Vine & Beverage Sciences, 523391 University of West Attica, 28 Ag. Spyridonos str, Egaleo 12243, Greece

**Keywords:** bee products, polyphenols, method validation, 4-hydroxybenzaldehyde, pinocembrin, HRMS

## Abstract

An innovative 4D targeted method was developed for the determination of 61 bioactive compounds in royal jelly (RJ) related to their health-promoting properties. The method, apart from high-resolution mass spectrometry, exploits the advantages of vacuum-insulated probe-heated electrospray ionization source (VIP-HESI), reducing thermal degradation, and trapped ion mobility spectrometry (TIMS), improving selectivity and compound identification. The optimization of VIP-HESI ionization parameters using experimental designs showed that the critical parameters were the capillary voltage as well as the probe gas flow rate and temperature. The LODs of the validated method ranged between 0.0013 and 0.090 μg g^–1^, the recoveries between 80 and 109%, while %RSDs were below 10%. The method was applied to 22 RJ samples, and 24 bioactive compounds were determined at concentrations between 0.098 μg g^–1^ (2-*cis*,4-*trans*-abscisic acid) and 18 μg g^–1^ (quinic acid), while eight compounds were identified for the first time. This method serves as a reliable analytical tool for a wide-scope chemical characterization of RJ.

## Graphical abstract

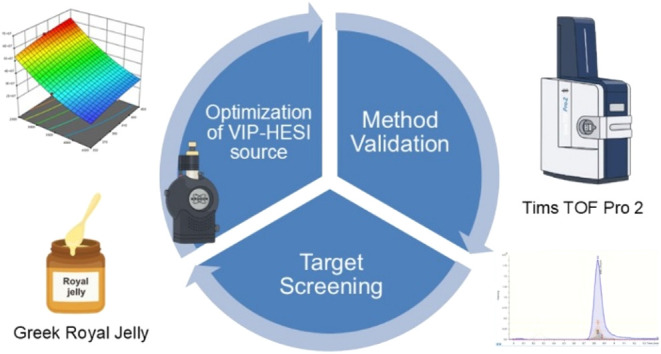

## 1 Introduction

Royal jelly (RJ) is a gelatinous, milky substance produced by the hypopharyngeal glands of the young honeybee workers (*Apis mellifera*), typically aged between 5 and 15 days old. It has a unique position in the beekeeping world, since it ensures the hive’s hierarchy. RJ is the food source for all honeybee larvae during the first 3 days following birth, and the exclusive food of the queen bee for her entire life, as its exceptionally complex composition offers the bee queen significant longevity and fertility compared to her worker counterparts.^1,2^

Although RJ’s composition and characteristics are affected by various factors, including honeybee species, geographical area, harvest time, and storage conditions,^3,4^ it consists mainly of water (60–70%), carbohydrates (7–18%), proteins (9–18%), and lipids (3–8%). It also contains free amino acids, minerals, vitamins, and polyphenols.^3^ Its pH value varies from 3.6 to 4.2, and its density is 1.1 g mL^–1^. Its color can range from white to yellow, and its odor and taste are lightly pungent.^3,4^

The harvest of RJ takes place when the larva in the queen cell is 3–4 days old. In this time frame, the supply of RJ is the largest and is of the highest grade in terms of both potency and purity. Although there are no official data on the production of RJ, it is estimated that the annual global production is a few thousand tons. China is the largest producer, representing about 60% of the world’s production. In Europe, RJ is mainly produced in Greece, Spain, France, and Italy.^5^

RJ has been used in medicine since antiquity for its health-beneficial properties. However, it is only in the last few decades that the interest in RJ has increased, as people are looking for new ways to protect and reinforce their immune system based on natural products, turning it into the most expensive of all bee products.^6^ Existing evidence proves that RJ has antioxidant, antimicrobial, antifungal, anti-inflammatory, and antibacterial activities.^7^ Today, RJ is recognized as a functional food, with recommended daily doses for adults typically ranging from 1 to 10 g.^8^ Moreover, RJ serves as a dietary supplement in the form of capsules, powders, or directly as a fresh or freeze-dried substance, but also in a plethora of pharmaceutical products as the main ingredient.^2^ RJ is also widely used in the field of cosmetics, since its use has been linked to skin health, antiaging, and wound healing.^6,9,10^

Some of RJ’s beneficial characteristics have been related to the high content of specific bioactive components, especially phenolic compounds. Phenolic compounds are plant secondary metabolites varying in structure from simple phenols to extremely complex polymeric compounds, all having in common at least one aromatic ring bound with one or more hydroxyl groups. More than 10,000 phenolic compounds have already been identified in plants, fruits, vegetables, tea, chocolate, and other foods.^11^

Although there is an increased interest in the existence of bioactive compounds in several foods, the number of studies regarding the extended chemical characterization of RJ’s bioactive profile, especially its phenolic content, remains limited. To date, reversed-phase high-performance liquid chromatography (HPLC) coupled to ultraviolet (UV)–visible absorbance detection has been applied for the determination of RJ’s phenolic compounds such as caffeic acid, ferulic acid, naringin, quercetin, and others.^12,13^ However, these studies present a low degree of certainty on the identification of the compounds, since they are based only on the retention time of standard solutions. They also exhibit limited selectivity, a low number of analytes, which reaches up to 12,^13^ and lack of validation data. Some of the inherent limitations of the HPLC-UV technique are partially addressed through the application of liquid chromatography coupled to mass spectrometry (LC-MS).^14–16^ Reversed-phase liquid chromatography with diode array detection coupled to electrospray ionization and time-of-flight mass spectrometry was used for the determination of eight flavonoid aglycone compounds (baicalein, chrysin, fisetin, hesperetin, kaempferol, naringenin, and quercetin) in honey samples and related products, including RJ. The identification was based on the retention time, the UV spectra, and the deprotonated ion of the analytes.^14^

In another work, where the effect of queen cell numbers on RJ’s production and quality was studied, reversed-phase and hydrophilic interaction liquid chromatography, coupled to high-resolution mass spectrometry (HRMS), consisting of a quadrupole and an Orbitrap, was applied. The methodology followed was rather a suspect screening for the general metabolic profiling of RJ, with only two findings on polyphenols (caffeic acid and chrysin) but without quantification or validation data.^15^ Finally, a validated method based on reversed-phase liquid chromatography coupled to Orbitrap was applied for the determination of approximately 50 polyphenols in RJ.^16^ Apart from RJ, HPLC coupled to HRMS has been applied for the accurate compound determination in other food matrices such as wine, olive oil, and milk.^17–19^

Based on the available data, it becomes obvious that there is an urgent need for the standardization of accurate, reliable, and robust analytical methods for polyphenols and bioactive compounds determination in RJ. Such methodologies will not only help to reveal the bioactive chemical profile of RJ, but they can also serve as a tool for ensuring its quality and authenticity, taking into consideration that RJ’s high nutritional and commercial value, along with its health-promoting properties, make it prone to adulteration.^20–22^

The aim of the present study is to take one step further in the determination of bioactive compounds in RJ by applying ultra-high-performance liquid chromatography (UHPLC) coupled to an ionization source consisting of a vacuum-insulated probe-heated electrospray ionization (VIP-HESI), followed by trapped ion mobility spectrometry (TIMS) and quadrupole time-of-flight (QTOF) mass spectrometry. This new 4D metabolomics methodology is applied for the first time for the mapping of RJ’s bioactive profile.

The heated electrospray ionization source (HESI) includes a heating device in the ESI probe body that heats the probe gas (N_2_) to temperatures between 200 and 600 °C. When the coaxial flow of heated nitrogen gas meets the generated aerosol emitted from the ESI capillary, the charged droplets evaporate more rapidly than when using ambient nitrogen, which is the case on the nonheated ESI, thereby increasing the ionization efficiency.^23,24^ This has been proven experimentally for some metabolites after comparison of the HESI source to an unheated ESI source during triple quadrupole MS experiments, in terms of absolute ion counts. However, it was noted that the noise also increased in most cases, but the overall signal-to-noise ratios were nevertheless improved. It is highlighted that the performance of the HESI source is compound-dependent and that certain compounds may decompose.^24^ Cutting-edge technology on ionization sources proposes the use of a vacuum-insulated probe (VIP) to address the thermal degradation of thermally labile compounds. Within the vacuum-insulated probe, the LC-eluent stream containing the compounds of interest is effectively shielded against heat by a vacuum barrier, reducing the heat transfer from the hot ceramic heater to the sample.^25^ Comparison of ESI and VIP-HESI source regarding LC-QTOF determination of mycotoxins in dust showed that the application of the VIP-HESI source in this complex matrix resulted in a significant increase in sensitivity for the majority of the investigated compounds, as the LODs were reduced by a factor of 2.5 on average.^26^

Applying TIMS, a variation of ion mobility spectrometry (IMS), ions are differentiated in the gas phase according to their charge, size, and shape.^27^ It provides high-resolution IMS by holding ions against the incoming gas stream using an electrostatic gradient tunnel. Utilizing the collision cross section values (CCS values), TIMS has been used to improve compound annotation, distinguish isomers and isobars, and increase the signal-to-noise (S/N) ratio in the MS spectrum.^28,29^ In addition to this, TIMS TOF, with the dual-TIMS analyzer, provides a nearly 100% duty cycle and three times larger ion capacity by storing ions in TIMS tunnel 1 and releasing them successively in TIMS tunnel 2.^30^ The incorporation of CCS values provided by TIMS, along with the retention time from LC, the *m*/*z* of the parent ion, and the *m*/*z* of the fragment ion(s), enables a 4D approach in metabolomic analysis.^31^

In the present study, a cutting-edge HRMS, UHPLC-VIP-HESI-TIMS-QTOF-MS targeted method was developed, optimized, and validated for the determination of bioactive compounds in RJ. Twenty-two RJ samples from various geographical regions all over Greece were analyzed to evaluate the applicability of this methodology, and a significant number of bioactive compounds were identified and quantified, showing that this protocol can be a powerful tool for comprehensive chemical characterization, quality assurance, and authenticity establishment of RJ.

## 2 Materials and Methods

### 2.1 Chemicals and Reagents

All reagents and standards used in this study were of high analytical purity. In particular, 2,5-dihydroxybenzoic acid (purity ≥99.0%), 3,4,5-trimethoxybenzoic acid (purity ≥99%), 3,4-dihydroxybenzoic acid (purity ≥97.0%), 4-hydroxybenzaldehyde (purity ≥98%), 4-hydroxybenzoic acid (purity ≥99%), acacetin (purity ≥97%), apigenin (purity ≥99%), benzoic acid (purity ≥99.5%), caffeic acid (purity ≥98.0%), catechin (purity ≥98%), catechol (purity ≥99%), chlorogenic acid (purity ≥95%), chrysin (purity ≥99.0%), cinnamic acid (purity ≥99%), diosmetin (purity ≥99%), epicatechin (purity ≥98%), eriodictyol (purity ≥98.0%), ethyl caffeate (purity ≥99.0%), ethyl gallate (purity ≥96.0%), ethyl vanillin (purity ≥98.5%), ferulic acid (purity ≥99%), fraxetin (purity ≥99%), fraxidin (purity ≥98.0%), hydroxytyrosol (purity ≥98%), kaempferide (purity ≥99%), luteolin (purity ≥97.0%), maslinic acid (purity ≥98%), myricetin (purity ≥98%), naringin (purity ≥95.0%), neohesperidin (purity ≥95.0%), *p*-coumaric acid (purity ≥98.0%), phloretin (purity ≥99%), phloridzin (purity ≥98.5%), pinobanksin (purity ≥99.0%), pinocembrin (purity ≥99.0%), pinoresinol (purity ≥95.0%), protocatechuic acid ethyl ester (purity ≥97%), quercetin (purity ≥95%), quinic acid (purity ≥99%), resveratrol (purity ≥99%), rosmarinic acid (purity ≥99.0%), rutin (purity ≥99%), sakuranetin (purity ≥98.0%), salicylic acid (purity ≥99.0%), scopoletin (purity ≥99%), sinapic acid (purity ≥98%), syringaldehyde (purity ≥98%), syringic acid (purity ≥98%), taxifolin (purity ≥85%), vanillic acid (purity ≥97%) and vanillin (purity ≥99%) were purchased from Sigma-Aldrich (St. Louis, MO). Galangin (purity ≥97%), gallic acid (purity ≥98%), genistein (purity ≥99%), hesperetin (purity ≥97%), naringenin (purity ≥97%), polydatin (purity ≥95%), and tyrosol (purity ≥98%) were obtained from Alfa Aesar (Karlsruhe, Germany). Kaempferol (purity ≥98%) and 2-*cis*,4-*trans*-abscisic acid (purity ≥98%) were purchased from Santa Cruz-Biotechnology (Santa Cruz, CA), while verbascoside (purity ≥99%) was purchased from HWI pharma services GmbH (Frankfurt, Germany). Finally, fraxin (purity ≥99%) was obtained from Extrasynthese (Genay, France).

LC-MS grade methanol (MeOH) was purchased from Merck (Darmstadt, Germany), while ammonium acetate with a purity of 99% was from Sigma-Aldrich (St. Louis, MO). A Milli-Q purification system (Millipore Direct-Q UV, Bedford, MA) was used to provide ultrapure water (18.2 MΩ cm^–1^). Regenerated cellulose syringe filters (RC filters, pore size 0.2 μm, diameter 15 mm) were purchased from Phenomenex (Torrance, CA).

Stock solutions of each compound mentioned above were prepared at a concentration of 1000 μg mL^–1^ in MeOH and stored at −20 °C in dark glass bottles. Working mix solutions at concentrations of 0.0050, 0.010, 0.025, 0.050, 0.10, 0.50, 1.0, 2.5, 5.0, and 10 μg mL^–1^ were then prepared by making appropriate dilutions of the standards in MeOH.

### 2.2 Royal Jelly Samples

A total of 22 fresh RJ samples were collected from beekeepers across different geographical regions around Greece. All samples were harvested in 2023 during the spring and summer seasons. They were stored in sealed glass containers in the freezer at −20 °C until analysis to decrease the possibility of quality deterioration due to light and temperature exposure. The detailed description of the geographical origin and harvest period of each sample is presented in Table S1.

### 2.3 Sample Preparation

For sample preparation, the protocol developed by Ma et al.^15^ was used with slight modifications. Particularly, 0.1 g of RJ sample was weighed in a centrifuge tube, and 4 mL of 80% aqueous methanol (v/v) was added. After vortexing for 5 min, samples were ultrasonicated for 20 min and then centrifuged at 10,000 rpm at 4 °C for 15 min. A volume of 1 mL of the supernatant was filtered through cellulose syringe filters directly into an HPLC vial.

### 2.4 UHPLC-VIP-HESI-TIMS-QTOF-MS Measurements

#### 2.4.1 UHPLC-VIP-HESI-TIMS-QTOF-MS System

The measurements of the current study were carried out with a UHPLC system (Elute LC series, Bruker Daltonics, Bremen, Germany) coupled to a VIP-HESI ionization source (Bruker Daltonics, Bremen, Germany). This ionization source has an active exhaust system, which eliminates recirculation of nebulized gases due to differential pressure between the chamber and the exhaust, reducing the chemical background during measurements of complex matrices. The VIP-HESI system was connected to a timsTOF Pro 2 mass spectrometer (Bruker Daltonics, Bremen, Germany), which combines TIMS-QTOF-HRMS.

#### 2.4.2 Chromatographic Conditions

Chromatographic separation was achieved using an Acclaim RSLC C18 column (2.1 × 100 mm, 2.2 μm) from Thermo Fischer Scientific (Waltham, MA), equipped with an Acquity UPLC BEH C18 VanGuard Pre-Column from Waters (Milford, MA), thermostated at 30 °C. The mobile phase consisted of (A) Milli-Q H_2_O:MeOH (90:10) and (B) MeOH, both containing 5 mM ammonium acetate. The gradient elution program started with 1% B with a flow rate of 0.2 mL min^–1^ for 1 min, increased to 39% in 2 min (flow rate: 0.2 mL min^–1^), and then increased to 99.9% (flow rate: 0.4 mL min^–1^) in the following 11 min. After being constant for 2 min (flow rate: 0.48 mL min^–1^), the initial conditions were restored within 0.1 min, kept for 3 min, and then the flow rate was decreased to 0.2 mL min^–1^. The injection volume was set to 2 μL.

#### 2.4.3 TIMS-QTOF Conditions

The timsTOF Pro 2 system operated in the broadband Collision-Induced Dissociation (bbCID) scan mode. A mass scan range of 30–1050 Da was selected. The range of inverse ion mobilities (1/*k*_0_) included values from 0.40 to 1.40 Vs cm^–2^. A duty cycle of 100% was achieved due to a dual-TIMS setup, given the fact that accumulation and ramp times were kept stable.^32,33^ Thus, in the present work, the accumulation and ramp times were both set at 100 ms. Prior to batch analysis, ion mobility, and *m*/*z* measurements were both externally calibrated using Agilent ESI Low Concentration Tune Mix (Agilent Technologies) [*m*/*z*, 1/*k*_0_: (301.9981, 0.6678 Vs cm^–2^), (601.9790, 0.8782 Vs cm^–2^)] in negative mode. The mobility calibration was performed using mobility values retrieved from the CCS compendium.^34^

#### 2.4.4 Target Screening

An accurate-mass target database was created and used for the identification and quantification of certain bioactive compounds, mainly phenolic compounds, in RJ samples. The database contained 61 compounds belonging to different classes including phenolic acids, lignans, stilbenes, phenolic aldehydes, and flavonoids. It is noted that although benzoic acid, quinic acid, and abscisic acid are nonphenolic compounds, they are included in the target database as they represent important bioactive compounds that have already been mentioned in bee products.^35–37^ The complete target screening database with information about the name of the analyte, the chemical class, the molecular formula, the retention time, the accurate masses of the principal and qualifier ions, the 1/*k*_0_, the CCS values, and the reference related to the presence of the analyte in bee products is presented in Table 1.

**Table 1:** Target Screening Database of Bioactive Compounds

analyte	chemical class	molecular formula	[M – H]^−^ *m*/*z*	retention time (min)	qualifier 1 *m*/*z*	qualifier 2 m/z	qualifier 3 *m*/*z*	1/*k*_0_ (Vs cm^–2^)	CCS (Å^2^)	refs
fraxidin	coumarin	C_11_H_10_O_5_	221.0455	4.8	190.9986	163.0037	135.0088	0.679	144.3	^39^
scopoletin	coumarin	C_10_H_8_O_4_	191.0350	4.8	176.0115	148.0166	120.0217	0.626	134.2	^40^
fraxin	coumarin glycoside	C_16_H_18_O_10_	369.0827	3.8	207.0299	192.0064	289.0729	0.854	177.4	^41^
acacetin	flavonoid	C_16_H_12_O_5_	283.0612	9.1	268.0377	283.0611	239.0350	0.797	167.3	^16^
apigenin	flavonoid	C_15_H_10_O_5_	269.0455	7.3	117.0346	151.0037	149.0244	0.749	157.6	^16^
catechin	flavonoid	C_15_H_14_O_6_	289.0718	3.7	245.0819	123.0452	125.0244	0.747	156.6	^42^
chrysin	flavonoid	C_15_H_10_O_4_	253.0506	8.7	63.0240	143.0502	209.0608	0.738	156.2	^16^
diosmetin	flavonoid	C_16_H_12_O_6_	299.0561	7.4	284.0326	256.0377	151.0037	0.818	171.3	^42^
epicatechin	flavonoid	C_15_H_14_O_6_	289.0718	4.2	245.0819	123.0452	125.0244	0.747	156.6	^43^
eriodictyol	flavonoid	C_15_H_12_O_6_	287.0560	6.0	135.0452	151.0037		0.784	164.5	^44^
fraxetin	flavonoid	C_10_H_8_O_5_	207.0299	4.6	192.0064	147.0452	164.0115	0.642	136.9	^45^
galangin	flavonoid	C_15_H_10_O_5_	269.0455	9.3	213.0557	169.0659	143.0502	0.740	155.7	^44^
genistein	flavonoid	C_15_H_10_O_5_	269.0455	6.7	63.0240	133.0295	159.0452	0.762	160.3	^16^
hesperetin	flavonoid	C_16_H_14_O_6_	301.0718	6.6	164.0115	151.0037	242.0585	0.826	172.9	^16^
kaempferide	flavonoid	C_16_H_12_O_6_	299.0561	9.5	284.0326	151.0037	107.0139	0.800	167.5	^40^
kaempferol	flavonoid	C_15_H_10_O_6_	285.0405	7.3	229.0506	185.0608	159.0451	0.766	160.8	^46^
luteolin	flavonoid	C_15_H_10_O_6_	285.0405	6.8	133.0295	151.0037	175.0401	0.765	160.4	^47^
myricetin	flavonoid	C_15_H_10_O_8_	317.0303	5.9	151.0037	178.9986	137.0244	0.797	166.4	^40^
naringenin	flavonoid	C_15_H_12_O_5_	271.0612	6.4	119.0502	151.0037	107.0138	0.774	162.7	^16^
phloretin	flavonoid	C_15_H_14_O_5_	273.0768	6.6	167.0350	123.0452	119.0502	0.776	163.1	^40^
pinobanksin	flavonoid	C_15_H_12_O_5_	271.0612	6.5	253.0495	197.0597	225.0546	0.752	158.1	^44^
pinocembrin	flavonoid	C_15_H_12_O_4_	255.0663	8.2	151.0037	213.0557	107.0139	0.748	157.8	^44^
quercetin	flavonoid	C_15_H_10_O_7_	301.0354	6.5	151.0037	178.9986	121.0295	0.781	163.5	^47^
sakuranetin	flavonoid	C_16_H_14_O_5_	285.0769	8.2	119.0502	165.0193	93.0346	0.806	169.1	^16^
taxifolin	flavonoid	C_15_H_12_O_7_	303.0510	4.6	125.0244	285.0404	153.0193	0.788	164.9	^44^
naringin	flavonoid glycoside	C_27_H_32_O_14_	579.1719	5.1	271.0641	151.0029	579.1728	1.045	214.2	^40^
neohesperidin	flavonoid glycoside	C_28_H_34_O_15_	609.1825	5.2	301.0736	609.1862	302.0755	1.105	226.0	^40^
phloridzin	flavonoid glycoside	C_21_H_24_O_10_	435.1297	5.3	273.0768	167.0350	179.0350	0.940	194.2	^40^
rutin	flavonoid glycoside	C_27_H_30_O_16_	609.1461	5.1	301.0354	331.0457	197.0457	1.136	232.5	^48^
pinoresinol	lignan	C_20_H_22_O_6_	357.1344	6.1	151.0401	136.0166		0.946	196.7	^40^
2-*cis*,4-*trans*-abscisic acid	organic acid	C_15_H_20_O_4_	263.1289	4.6	204.1156	219.1391	151.0765	0.791	166.6	^44^
benzoic acid	organic acid	C_7_H_6_O_2_	121.0295	4.5	77.0397	93.0346		0.528	117.2	^40^
quinic acid	organic acid	C_7_H_12_O_6_	191.0561	1.3	85.0295	127.0401	173.0455	0.627	134.4	^47^
catechol	phenol	C_6_H6O_2_	109.0295	4.0	91.0189	81.0346		0.502	112.6	^49^
2,5-dihydroxybenzoic acid (gentisic acid)	phenolic acid	C_7_H_6_O_4_	153.0193	2.1	108.0217	109.0295		0.567	123.4	^44^
3,4,5-trimethoxy-benzoic acid (eudesmic acid)	phenolic acid	C_10_H_12_O_5_	211.0612	3.2	152.0479	167.0714	137.0244	0.715	152.3	^44^
3,4-dihydroxybenzoic acid (protocatechuic acid)	phenolic acid	C_7_H_6_O_4_	153.0193	1.3	108.0212	125.0243	93.0340	0.564	122.8	^47^
4-hydroxybenzoic acid	phenolic acid	C_7_H_6_O_3_	137.0244	1.3	93.0346	65.0397		0.541	118.7	^46^
caffeic acid	phenolic acid	C_9_H_8_O_4_	179.0350	2.1	135.0452	119.0501	134.0374	0.611	131.5	^47^
chlorogenic acid	phenolic acid	C_16_H_18_O_9_	353.0878	2.3	191.0561	135.0452	179.0350	0.821	170.8	^47^
cinnamic acid	phenolic acid	C_9_H_8_O_2_	147.0452	4.7	121.0269	137.0241		0.578	126.3	^44^
ferulic acid	phenolic acid	C_10_H_10_O_4_	193.0506	2.6	134.0373	178.0272	133.0295	0.648	138.8	^16^
gallic acid	phenolic acid	C_7_H_6_O_5_	169.0142	1.3	125.0244	69.0346	97.0295	0.573	123.8	^47^
*p*-coumaric acid	phenolic acid	C_9_H_8_O_3_	163.0401	2.3	119.0502	93.0335		0.596	129.1	^47^
rosmarinic acid	phenolic acid	C_18_H_16_O_8_	359.0772	4.1	161.0233	197.0444	179.0338	0.828	172.2	^43^
salicylic acid	phenolic acid	C_7_H_6_O_3_	137.0244	3.3	93.0346	65.0397		0.538	118.1	^44^
sinapic acid	phenolic acid	C_11_H_12_O_5_	223.0612	2.8	193.0142	149.0244	121.0295	0.696	147.8	^40^
syringic acid	phenolic acid	C_9_H_10_O_5_	197.0455	1.3	123.0088	166.9986	182.0221	0.643	137.7	^44^
vanillic acid	phenolic acid	C_8_H_8_O_4_	167.0350	1.3	108.0217	152.0115	123.0452	0.594	128.4	^44^
verbascoside	phenolic acid derivative	C_29_H_36_O_15_	623.1981	4.7	161.0244	135.0452	179.0350	1.086	222.3	^42^
4-hydroxybenzaldehyde	phenolic aldehyde	C_7_H_6_O_2_	121.0284	4.3	108.0214	93.0349		0.524	116.4	^50^
ethyl vanillin	phenolic aldehyde	C_9_H_10_O_3_	165.0557	5.2	136.0166	108.0217	92.0262	0.607	131.4	^51^
syringaldehyde	phenolic aldehyde	C_9_H_10_O_4_	181.0506	5.5	151.0037	123.0088	166.0271	0.641	137.8	^40^
vanillin	phenolic aldehyde	C_8_H_8_O_3_	151.0401	4.6	136.0166	108.0217	92.0268	0.574	125.2	^44^
ethyl caffeate	phenolic ester	C_11_H_12_O_4_	207.0652	6.6	135.0451	133.0296	161.0243	0.688	146.8	^40^
ethyl gallate	phenolic ester	C_9_H_10_O_5_	197.0445	4.9	125.0247	169.0140		0.648	138.7	^42^
protocatechuic acid ethyl ester	phenolic ester	C_9_H_10_O_4_	181.0495	5.5	109.0296	153.0194		0.632	136.1	
hydroxytyrosol	phenylethanoid	C_8_H_10_O_3_	153.0557	3.3	123.0451	95.0502	108.0210	0.591	128.7	^39^
tyrosol	phenylethanoid	C_8_H_10_O_2_	137.0608	4.1	119.0502	93.0346	107.0502	0.581	127.6	^42^
polydatin	stilbenoid	C_20_H_22_O_8_	389.1231	4.6	227.0714	197.0360	124.0238	1.031	213.7	^52^
resveratrol	stilbenoid	C_14_H_12_O_3_	227.0714	5.3	143.0502	185.0608	159.0815	0.743	157.6	^43^
maslinic acid	terpenoid	C_30_H_48_O_4_	471.3480	12.4	423.3265	227.2028	269.0470	1.084	223.4	^53^

The identification criteria were the following: retention time tolerance lower than ±0.2 min, mass error of the precursor and qualifier ions less than 2 mDa, isotopic fit less or equal to 50 mSigma (Bruker mSigma is a measure of fitness between the measured and the theoretical isotopic pattern), the existence of at least two qualifier ions and ΔCCS less or equal to 2%.^38^ Target screening workflow was conducted using DataAnalysis 5.3 and TASQ 2023 software (Bruker Daltonics, Bremen, Germany). Additionally, the Mobility Calculator and SmartFormula Manually tools, available in this software, were also employed to calculate the CCS values of the target compounds.

Matrix-matched calibration curves were used to quantify the identified target analytes. Matrix-matched calibration curves were preferred instead of the external standard calibration curves due to the existence of the matrix effect that was depicted following the validation experiments.

### 2.5 VIP-HESI System Optimization

The operating parameters of the VIP-HESI source were optimized to obtain the maximum sensitivity and minimum matrix effect for the target analytes in RJ extracts. The effect of seven parameters on the ionization of the target analytes was studied: (i) the position of the sprayer, (ii) the nebulizer gas pressure (N_2_), (iii) the capillary voltage, (iv) the probe gas flow rate (N_2_), (v) the probe gas temperature, (vi) the dry gas flow rate (N_2_), and (vii) the dry gas temperature in negative ionization mode.

For the optimization experiments, the RJ 22 sample was used, which was found to contain lower concentrations of the target compounds in comparison to the rest of the samples during the preliminary experiments. The treated RJ sample was spiked with the target analytes, presented in Section 2.1, at a concentration of 1.0 μg g^–1^, and it was analyzed by UHPLC–TIMS–QTOF-MS as described in Section 2.4 under the different HESI conditions, as described below. The monitored parameters (responses) were the peak areas of the deprotonated ion [M – H]^−^ of 6 selected target analytes, namely apigenin, chrysin, pinobanksin, quercetin, scopoletin, and 2-*cis*,4-*trans* abscisic acid, which differ in *m*/*z*, retention time, CCS values, and sensitivity.

As a first step of the optimization procedure, all of the possible combinations of the position of the sprayer and the nebulizer gas pressure were tested. The sprayer can move on the *y*-axis, which is vertical to the axis of the entrance cone leading to the spectrometer, and it can take three possible positions: Up (+4 mm), Middle (0 mm), and Down (−4 mm), as it is shown in the schematic diagram of Figures 1 and S1 in the Supporting Information. Similarly, the three possible positions on the *x*-axis, which is parallel with the axis of the entrance cone leading to the spectrometer, are Near (+3 mm), Middle (0 mm), and Far (−3 mm). Combining the information for the *x* and *y* axes, all nine possible probe positions tested are shown in Figure 1, and they are described as Far Up, Far Middle, Far Down, Middle Up, Middle Middle, Middle Down, Near Up, Near Middle, and Near Down. The tested values for the nebulizer gas pressure were 1.5, 2.0, 2.5, 3.0, and 3.5 bar. All nine sprayer’s positions were matched with all five nebulizer gas pressures, and the optimum combination was selected.

**Figure 1:**
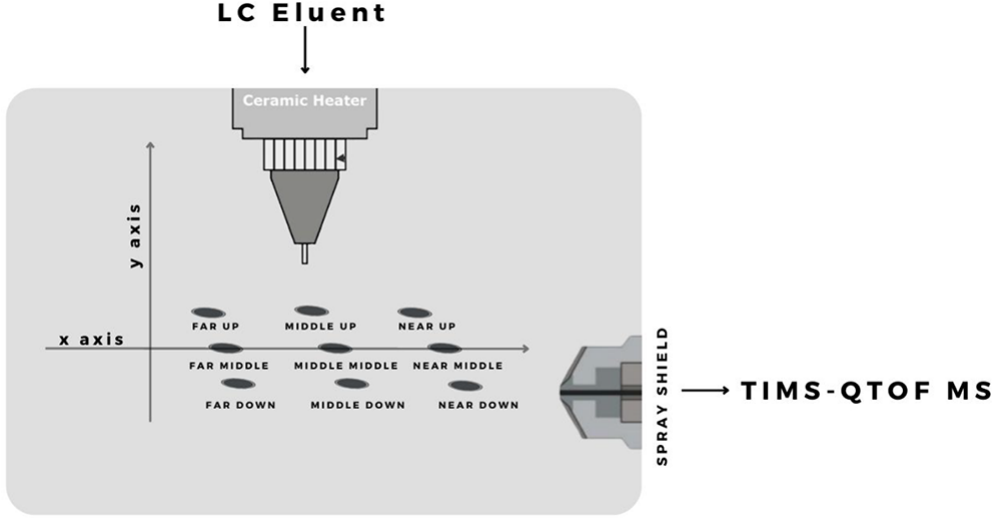
Schematic diagram of all the possible sprayer’s positions in the ionization source.

#### 2.5.1 Plackett–Burman Design

Subsequently, a multivariate optimization of the other five parameters of the VIP-HESI source was conducted based on a screening Plackett–Burman design in order to identify the critical factors that affect the signal response of the selected compounds. Plackett–Burman design requires *n* + 1 experiments, where n is the number of variables. When the number of variables is lower than 11, which is the minimum for the model, dummy variables are added.^54^ The studied parameters were the capillary voltage (A), the dry gas flow (B), the dry gas temperature (C), the probe gas flow (D), the probe gas temperature (E), and six dummy variables (F–K). Three levels were tested for each parameter, as shown in Table 2, selected according to the proposed values by the manufacturer and preliminary experimental results of the present study. Therefore, a 16-run Plackett–Burman design, 12 experiments and four center points, presented in detail in coded levels in Table S2 in the Supporting Information, was conducted. The four center points were used to explore whether there is curvature in the model and to add this information to the estimation of repeatability. The effect of variables on the obtained signals is described by the first-order polynomial equation as follows:

1 $$Y_{i}=\beta ο+\beta_{1}A+\beta_{2}B+\beta_{3}C+\beta_{4}D+\beta_{5}E$$

where *Y_i_* (*i* = 1–6) stands for the response as peak area of each of the tested compounds, β_0_ stands for the intercept, β*_i_* the regression coefficients, and A, B, C, D, and E stand for the independent factors. The critical factors that significantly affect the response *Y* at a confidence level of 95% (*p* < 0.05) were identified.^55–57^

**Table 2:** Levels of the Variables Tested in Plackett–Burman Design

variable	symbol	(level) experimental value		
		(−1)	(0)	(+1)
capillary voltage (V)	A	2500	3500	4500
dry gas flow rate (L min^–1^)	B	8	9	10
dry gas temperature (°C)	C	230	240	250
probe gas flow rate (L min^–1^)	D	3	4	5
probe gas temperature (°C)	E	350	400	450
dummies	F–K	0	0	0

#### 2.5.2 Box–Behnken Design

After identifying the critical variables, a response surface methodology was followed to investigate the possible interactions between them and conclude on their optimum values. Therefore, a Box–Behnken experimental design was applied with the experimental values, summarized in Table 2, for the three critical parameters, which were the capillary voltage, the probe gas flow rate, and the probe gas temperature. A total of 16 experiments, presented in detail in Table S3 of the Supporting Information, were proposed by the Box–Behnken design for the adequate description of the model. A multiple regression analysis was performed to obtain the coefficients, and the equation was used to estimate the response. In particular, in a system involving three significant variables A, B, and C, the mathematical relationship of the response to these variables can be fitted by the quadratic (second-degree) polynomial equation:

2 $$Y_{i}=\beta_{0}+\beta_{1}A+\beta_{2}B+\beta_{3}C+\beta_{12}\mathrm{AB}+\beta_{13}\mathrm{AC}+\beta_{23}\mathrm{BC}+\beta_{11}A^{2}+\beta_{22}B^{2}+\beta_{33}C^{2}$$

where *Y_i_* (*i* = 1–6) stands for the response as peak area of each of the tested compounds, β_0_ is the constant, β_1_, β_2_, and β_3_ are linear coefficients, β_12_, β_13_, and β_23_ are interaction coefficients between the three factors, β_11_, β_22_, and β_33_ are quadratic coefficients. The statistical significance of the coefficients in the regression equation was investigated by the analysis of variance (ANOVA). The fitness of the polynomial model equation to the responses was evaluated with the coefficients of *R* ^2^, and the lack of fit was evaluated using F-test.^58,59^

The statistical analysis for the evaluation of the results and their potential interferences was conducted with Design-Expert 13.0 software (Stat-Ease, Inc., Minneapolis, MN).

### 2.6 Method Validation

Validation of the optimized analytical method is essential to ensure that it is appropriate for its intended use. The method was validated in terms of linearity, limit of detection (LOD), limit of quantification (LOQ), matrix effect, and accuracy through trueness and precision. For the validation experiments, matrix-matched solutions were prepared at concentrations of 0.0050, 0.010, 0.025, 0.050, 0.10, 0.50, 1.0, 2.5, 5.0, and 10 μg mL^–1^ by spiking the corresponding amount of the standard solutions in an RJ 22 extract.

Linearity was evaluated with matrix-matched calibration curves. The curves were created by least-squares linear regression analysis of the chromatographic peak area of each analyte versus concentration, and the squared regression coefficients (*R* ^2^) were determined.

The LODs and LOQs were calculated as the concentration for which the signal-to-noise ratios were 3 and 10, respectively.

Matrix effects were determined using eq 3, where “Area chromatogram mm” is the response in the matrix-matched standard, “Area chromatogram unspiked sample” is the analyte’s response in the untreated sample, and “Area chromatogram standard” is the analyte’s response in a standard solution prepared in solvent.
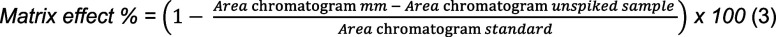


Three replicates of a spiked sample at three different concentrations (0.25, 1.0, and 5.0 μg g^–1^) were conducted on the same day under the same conditions to assess the method’s repeatability (intraday precision), expressed as % relative standard deviation (RSD %, *n* = 3), while for the interday precision, the same analysis was conducted in triplicate under the same conditions for two consecutive days.

Trueness was expressed as the recovery percentage at three concentration levels (0.25, 1.0, and 5.0 μg g^–1^). It was calculated as the ratio of the peak area of the spiked sample to the peak area of the corresponding matrix-matched standard.

## 3 Results and Discussion

### 3.1 Optimization of VIP-HESI Parameters

#### 3.1.1 Optimization of Sprayer’s Position and Nebulizer Gas Pressure

The obtained peak areas of the target analyte chrysin for the different sprayer positions under the different nebulizer gas pressures are presented in Figure 2. It was observed that, regarding the *y*-axis, which is vertical to the entrance of the spectrometer, the Up positions resulted in higher peak areas compared to the respective Down positions. The Up positions provide a longer distance for the ions to enter the spectrometer area, and potentially a larger amount of matrix ions failed to enter the spectrometer, reducing the matrix effect. On the contrary, on the *x*-axis, which is parallel with the entrance of the spectrometer, the Near positions gave higher peak areas than the Far ones. This could be attributed to a limited number of target ions reaching the entrance of the spectrometer when the sprayer was placed in the Far position. This observation regarding this dimension of the sprayer’s (probe) position was in accordance with previous work on the optimization of ESI parameters on Diuron, Iragarol, and their degradation products.^60^ Similar results were obtained for all of the tested analytes. The corresponding bar charts for apigenin, pinobanksin, quercetin, scopoletin, and 2-*cis*,4-*trans* abscisic acid are presented in Figure S2 in the Supporting Information, and for chrysin in Figure 2. In all cases, the optimum sprayer position was Near Up for all of the tested nebulizer gas pressures.

**Figure 2:**
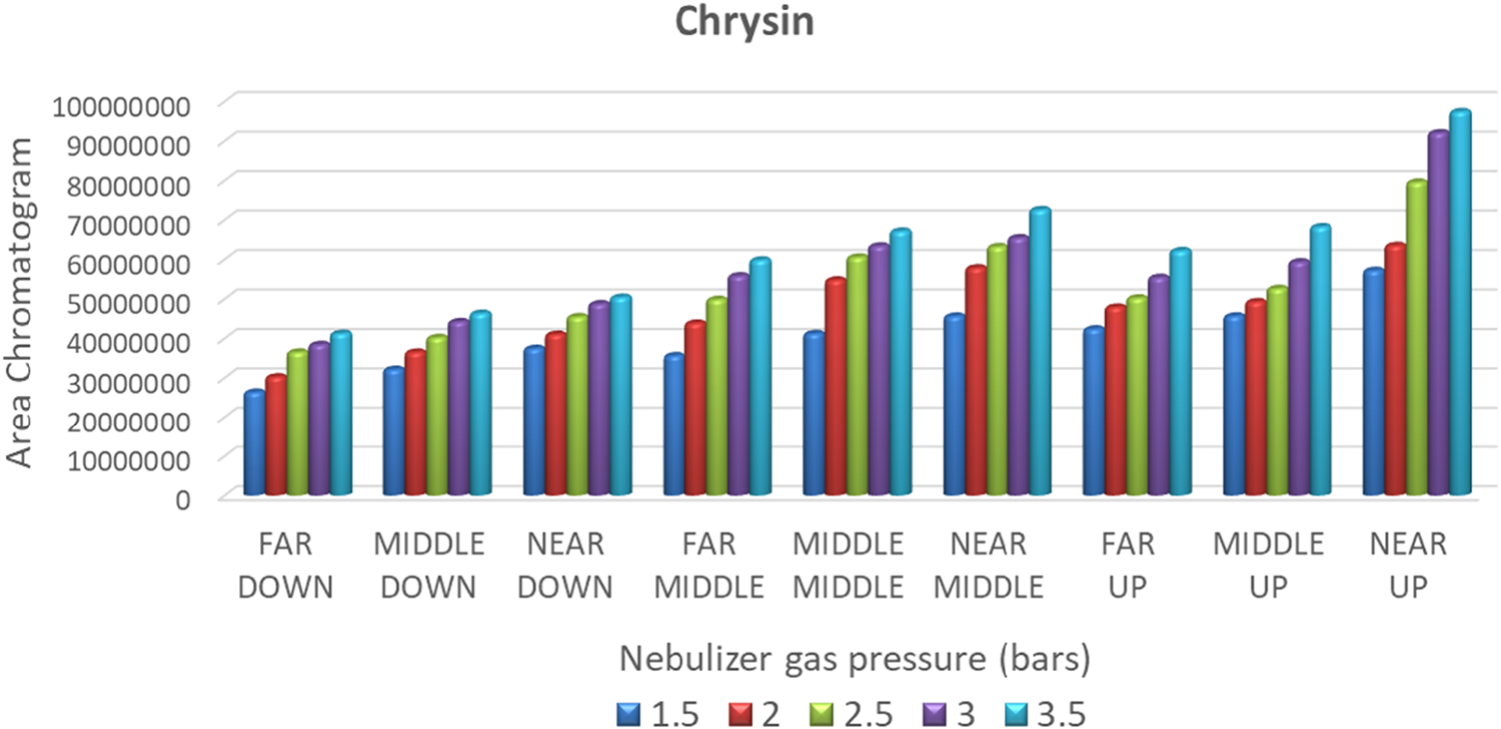
Bar chart of the peak area of chromatograms as a function of nebulizer gas pressure (bars) in different sprayer positions for Chrysin.

The comparison of the different nebulizer gas pressures on the obtained signal of chrysin is illustrated in Figure 2. It is shown that the higher the gas pressure, the higher the signal intensity for all the tested sprayer’s positions. The same results were obtained for all of the tested compounds (Figure S2 in the Supporting Information). Therefore, it can be concluded that a gas pressure of 3.5 bar was necessary to generate a proper mist of charged droplets. Summarizing the results of this set of experiments, it is concluded that the maximum sensitivity in terms of chromatographic peak areas is achieved with Near Up position and nebulizer gas pressure set at 3.5 bar.

#### 3.1.2 Multivariate Optimization of VIP-HESI Parameters

##### 3.1.2.1 Plackett–Burman Design

The results of the Plackett–Burman design for six representative compounds, apigenin, chrysin, pinobanksin, quercetin, scopoletin, and 2-*cis*,4-*trans* abscisic acid, in the form of Pareto charts presenting the *t*-value of IEffectI for each of the studied parameters are illustrated in Figure 3. The *t*-value limit is represented with a black threshold line and the more strict Bonferroni limit with a red threshold line. The variables with *t*-values of IEffectI beyond the *t*-value limit were considered statistically critical for the obtained signal at a confidence level of 95%. It is shown that the critical parametres that affect significantly the peak area of the tested compounds were the capillary voltage, the probe gas flow rate and the probe gas temperature, in descending order, with all three factors having *p*-values <0.0001 for most of the analytes, while dry gas flow (parameter B) and dry gas temperature (parameter C) were considered not statistically significant at confidence level 95% since their *t*-value of IEffectI was below the *t*-value limit and the Bonferroni limit.

**Figure 3:**
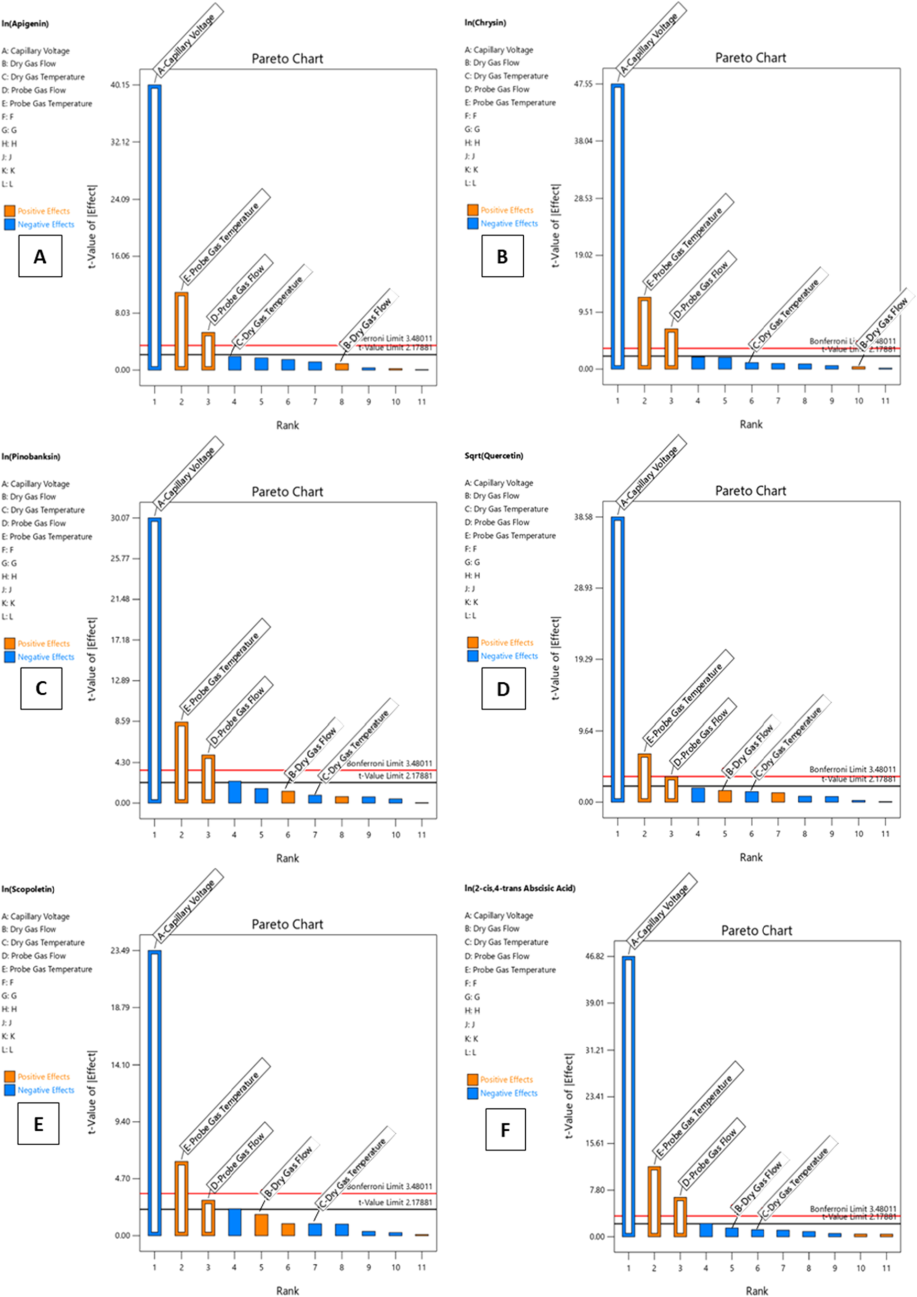
Pareto chart for (A) Apigenin, (B) Chrysin, (C) Pinobanksin, (D) Quercetin, (E) Scopoletin, and (F) 2-*cis*,4-*trans* Abscisic acid.

In more detail, the capillary voltage was critical for all the tested compounds, having a negative effect, which means that the higher the capillary voltage, the lower the obtained signal. The significance of the effect of the capillary voltage on the negative ionization of small organic compounds has been investigated in several studies, and controversial findings have been reported. For example, capillary voltage was not a critical factor for the electrospray negative ionization of 8-prenylnaringenin, zearalenone, α- and β-zearalenol,^61^ nor for the electrospray negative ionization of the flavonoids hesperidin and naringenin,^62^ whereas it appeared to have a significant negative effect on the sensitivity of several organic contaminants.^63^

The second critical parameter was the probe gas temperature, having a positive effect for all of the tested compounds, with quercetin having the highest positive effect (Table S4). The probe gas flow rate was identified as the third critical parameter for most of the tested compounds, which also presented a positive effect. The probe gas was a heated gas (N_2_) that met the generated aerosol at the end of the ESI capillary and contributed to the efficient desolvation process without thermal degradation of the fragile compounds. Therefore, the tested compounds appeared to require relatively intense conditions for their desolvation under the specific chromatographic conditions.

The detailed results of ANOVA (sum of squares, mean square, *F*-value, *p*-value, effect, regression coefficient, and standard error) for all the analytes are summarized in Tables S4 and S5, and the regression equations and the corresponding *R* ^2^ of the analytes are shown in Table S6 in the Supporting Information. It was observed that for the majority of the analytes, the *R* ^2^ values are higher than 0.98, demonstrating the good fit of the linear models.

In summary, the factors capillary voltage, probe gas flow rate, and probe gas temperature were considered critical and were studied further. On the contrary, the dry gas flow rate and the dry gas temperature were considered not critical. The selected numerical value of the dry gas flow rate was 10 L min ^–1^ on the basis that it had a positive effect on the obtained signal for most of the analytes, although it was not statistically significant. Similarly, the selected numerical value of the dry gas temperature was 230 °C based on its negative effect on the obtained signal for all the analytes, although not statistically significant.

##### 3.1.2.2 Box–Behnken Design

Based on the results from the screening Plackett–Burman design, a response surface methodology was applied with a Box–Behnken experimental design. Box–Behnken design, incorporating the three significant parameters, capillary voltage, probe gas flow, and probe gas temperature, was designed, while the dry gas flow rate and temperature were kept stable as described above. Results for the six representative analytes are shown in Table S7. ANOVA was applied to assess the effects of each parameter. Parameters with a *p*-value below 0.05 indicated that their effect is statistically significant on the obtained peak areas of the measured compounds.

It was observed that the results of Box–Behnken experimental design validate the results of the Plackett–Burman design since for all analytes, capillary voltage had very high *F*-values, and very low *p*-values (<0.0001), thus proving its statistical significance at a confidence level of 95%. Similarly, the probe gas flow rate and the probe gas temperature showed a high impact, although to a relatively lower extent compared to capillary voltage, with *p*-values <0.05.

Furthermore, with the Box–Behnken experimental design, it was possible to investigate the interaction effects between the three studied parameters. It was observed that the interaction between capillary voltage and probe gas flow rate (AB) was not significant for all of the compounds except for scopoletin, while the joint effect of capillary voltage and probe gas temperature (AC) was found to be significant for chrysin, pinobanksin, and scopoletin. Chrysin, as well as other flavonoids, are prone to oxidation, and therefore, high voltages may compromise the signal. The interaction between the probe gas flow rate and temperature (BC) was not statistically significant for any of the tested compounds.

Quadratic terms, A^2^, B^2^, and C^2^, had varying levels of significance, depending on the compound under investigation. For instance, apigenin’s A^2^ term (*p*-value: 0.0002) indicated a significant nonlinear capillary voltage effect. The B^2^ and C^2^ terms had lesser significance, with *p*-values of 0.0849 and 0.0653, respectively, indicating that the probe gas flow and temperature had weaker nonlinear effects. However, quercetin had substantial quadratic effects for all three terms, with A^2^ (*p*-value: 0.0028), B^2^ (*p*-value: 0.0244), and C^2^ (*p*-value: 0.0259). Furthermore, the *p*-values for A, B, and C showed that this compound had substantial nonlinear connections with capillary voltage, probe gas flow, and temperature.

In all cases, *R* ^2^ exceeded 0.99, with chrysin having the lowest *R* ^2^ value of 0.990. All models had strong adjusted and predicted *R* ^2^ values, indicating their reliability and robustness, like in the case of scopoletin with an adjusted *R* ^2^ of 0.9990 and a predicted *R* ^2^ of 0.997. This indicated that the model explained the variance in the data while maintaining a high predicted accuracy.

Figure 4 depicts response surface plots that demonstrate the interaction effects and trends among the key variables (capillary voltage, probe gas flow rate, and probe gas temperature) and the response factors of chysin. In addition, Figure S3 shows the response surface plots for five more investigated bioactive compounds. Τhe curved surface for chrysin indicated the impact of nonlinear correlations and interactions between the variables (Figure 4, plots A and B). The same applied for scopoletin, or 2-*cis*,4-*trans* abscisic acid, in Figure S3 in plots D_1_, D_2_, E_1_, and E_2_. However, for pinobanksin, the flat surface of the figure implied a linear relation between the variables in both plots B_1_ and B_2_.

**Figure 4:**
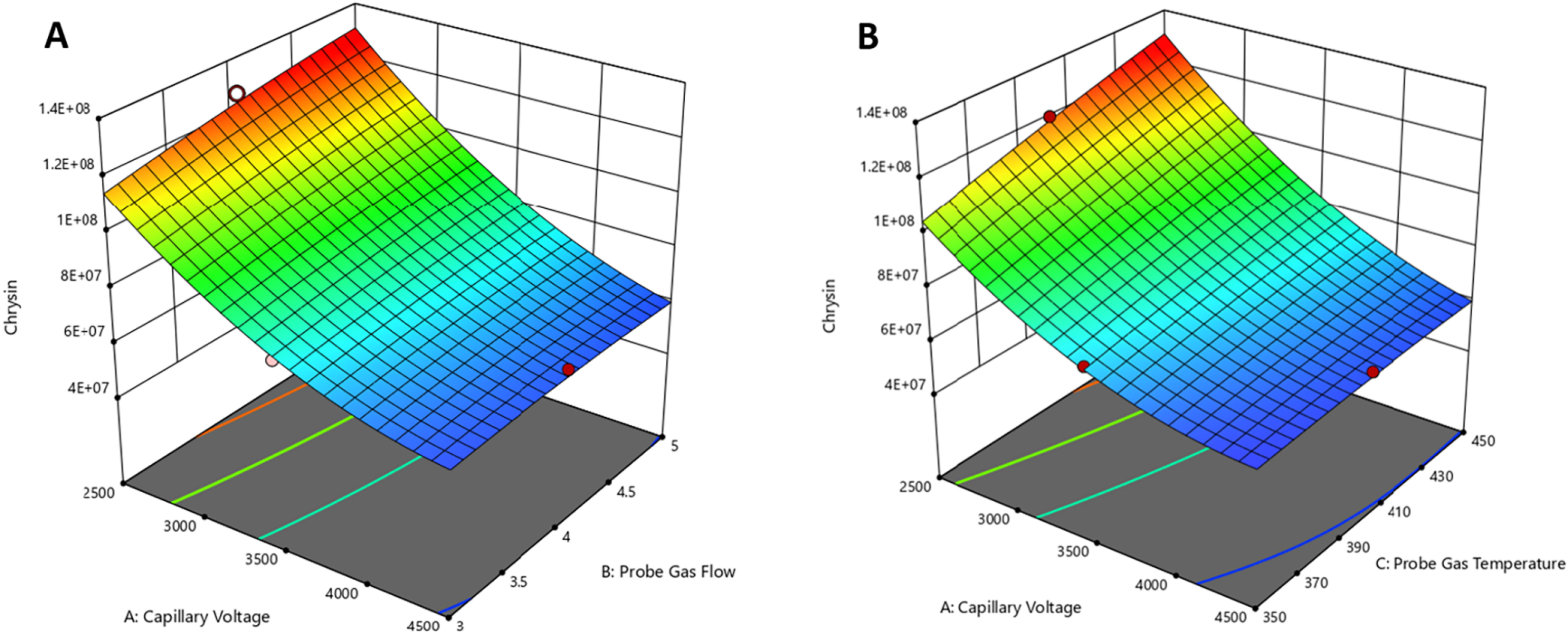
Response surface plots between (A) capillary voltage and probe gas flow and (B) capillary voltage and probe gas temperature for Chrysin.

Color changes were used to represent the gradient of the response variable. The red zones in the plots correspond to the highest areas of the chromatograms, indicating that the conditions were optimal. Stronger effects of the variables were presented with steeper gradients. For chrysin, plot A (Figure 4), which presents capillary voltage and probe gas flow, shows that the optimal conditions were met when the capillary voltage was 2500 V and the probe gas flow rate was 5 L min^–1^. In the plot comparing capillary voltage and probe gas temperature, shown in part B (Figure 4), the red zone was mainly located at a capillary voltage of 2500 V and a probe gas temperature of 450 °C. This combination provided the highest chromatographic area, indicating the most effective conditions for analysis. A consistency of these optimal conditions between different analytes was observed, as shown in Figure S3. Each analyte had the same red zone profile, indicating that overall, the capillary voltage of 2500 V, the probe gas temperature of 450 °C, and the probe gas flow rate of 5 L min^–1^ were the optimum values according to the applied experimental design. Figure S4 illustrates the contour plots of the desirability between these parameters.

#### 3.1.3 Optimum VIP-HESI Conditions

Summarizing the results, the final optimized protocol for the VIP-HESI source, operating in negative ionization mode, was as follows: capillary voltage, 2500 V; probe gas flow rate, 5 L min^–1^; probe gas temperature, 450 °C; dry gas flow rate, 10 L min^–1^; and dry gas temperature, 230 °C. The end plate offset was set at 500 V.

### 3.2 Method Validation

#### 3.2.1 Validation Results of the Optimized Methodology

The detailed validation results can be found in Tables S8 and S9. In Table S8, the correlation coefficient, LODs, LOQs, matrix effect, and recovery evaluated at 3 concentration levels are presented. Specifically, good linearity was achieved as all analytes had correlation coefficients higher than or equal to 0.98. Furthermore, 50 out of 55 analytes reached *R* ^2^ ≥ 0.990. LODs ranged from 0.0013 μg g^–1^ (phloridzin and 2-*cis*,4-*trans*-abscisic acid) to 0.090 μg g^–1^ (salicylic acid). Regarding the matrix effect, 34 compounds had a negative matrix effect, indicating ion enhancement, whereas 21 had a positive matrix effect, indicating ion suppression. Since 44 out of 55 compounds demonstrated a matrix effect greater than ±20%, it was deemed necessary to prepare matrix-matched calibration curves to compensate for ion suppression or enhancement and accurately quantify the bioactive compounds. The recovery results were all between 80 and 110%, ranging from 80 to 109%. The repeatability and intermediate precision of the method were also evaluated, and the results are presented in Table S9. Regarding repeatability, all the results of the RSD% were below 10%, varying between 0.82 and 7.4%, while in intermediate precision, all analytes had RSD% lower than 15%, except for hydroxytyrosol at the high level, which exhibited RSD% equal to 18. The satisfactory validation results for 55 compounds indicate the potential of the developed method for the determination of the bioactive profile of RJ.

#### 3.2.2 Comparison of the Validated Methodology with Previous Studies

Overall, the validated method in the present study offers several advantages in exploring the phenolic content of RJ. To date, there are published studies in which individual phenolic compounds were reported in RJ samples following the application of LC with UV detection.^12,13^ However, the analytical protocols of these works were not validated, lacking important information on the methods’ performance.

Among the validated methods, a limited number of criteria were included in the validation design. For example, in a study on the individual phenolic content of RJ,^14^ only recovery was estimated in the validation procedure without providing information about LODs, LOQs, repeatability, intermediate precision, and matrix effect, factors that were thoroughly examined in the present work. In addition, while the recovery of this study was supported using 8 different phenolic compounds, the present work included a significantly wider number of analytes, 55 in total.

A significant study, related to the phenolic profile of Spanish RJ, using LC-HRMS involved a slightly decreased number of compounds, 52 in total.^16^ LODs of this work ranged from 5 to 50 μg kg^–1^. The authors noted that LODs indicated the minimum concentration for which the mass error was below 5 ppm for the characteristic ion [M + H]^+^ or [M – H]^−^, while LOQs represented the minimum concentration for which the method was linear. In contrast, in this validated method, the LODs were determined by estimating the concentration corresponding to a signal-to-noise ratio of ≥3, and LOQs to a signal-to-noise ratio of ≥10. Following this approach, the range of LODs was from 0.0013 to 0.090 μg g^–1^ and LOQs from 0.0042 to 0.30 μg g^–1^, respectively. In terms of repeatability and intermediate precision, the present study achieved values <10 and <15%, respectively, while the corresponding values in the validated method from López-Gutiérrez et al. were <15 and <17%, respectively. However, it is noteworthy that the evaluated levels were different between these two studies. López-Gutiérrez et al. evaluated repeatability and intermediate precision at concentrations equal to 0.5, 1, and 2 μg g^–1^, whereas the present study covered a wider range with levels equal to 0.25, 1, and 5 μg g^–1^. Regarding recoveries, the obtained values according to López-Gutiérrez et al. varied from 68 to 108%, while in the present study, they were between 80 and 109%.

Taking into consideration the above, it becomes obvious that the existing analytical workflows for the determination of the phenolic and bioactive contents of RJ are limited. Even more limited studies include validation criteria. This study involved a high number of target compounds to examine LODs, LOQs, matrix effect, repeatability, intermediate precision, and recovery. The obtained results were satisfactory compared to those of other studies, strengthening the reliability and potential of the method for the analysis of the phenolic profile of RJ.

### 3.3 Occurrence and Concentration Levels of Bioactive Compounds in Royal Jelly Samples

A total of 24 phenolic compounds were identified and quantified using the optimized UHPLC-VIP-HESI-TIMS-QTOF-MS method in the 22 RJ samples, and the identification criteria are described in Section 2.4.4. A representative example of the identification procedure for chrysin using the TASQ 2023 software is shown in Figure 5. Specifically, in sample RJ 7, the detailed ion results of chrysin (*m*/*z* 253.0506) and the CCS values, Δ*m*/*z* [mDa], ΔRT [min], mSigma, *m*/*z* Score, RT score, and CCS score are presented. The extracted ion chromatogram (EIC) of the same analyte and the qualifier ions are shown at retention time (RT = 8.8 min). At last, the extracted ion mobilogram (EIM) of the compound is presented with 1/*k*_0_ = 0.74, and a CCS value of 156.33. The same procedure was applied for all of the positive findings. The area of the EIC was used to quantify target compounds with the use of matrix-matched curves.

**Figure 5:**
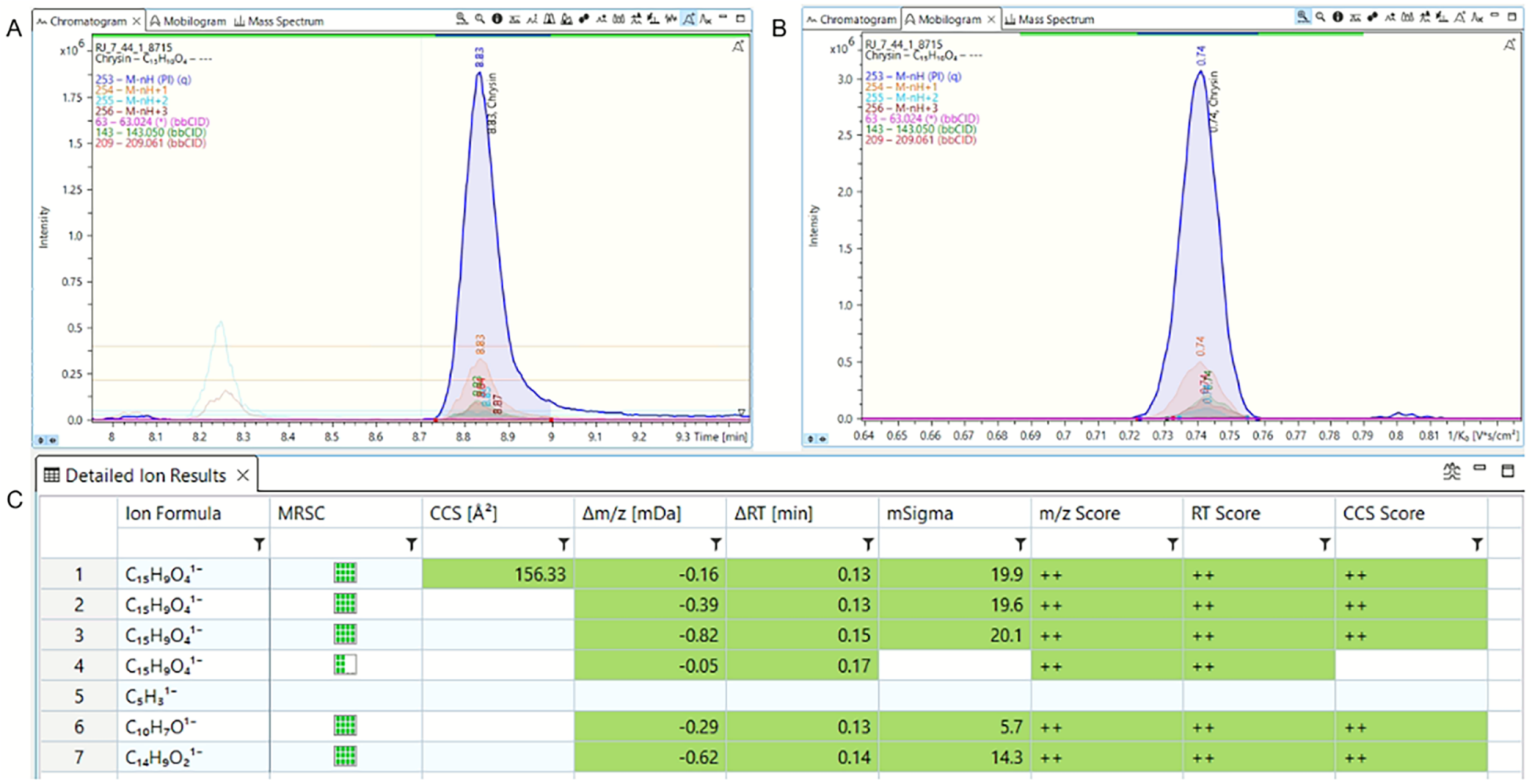
Identification workflow of chrysin in sample RJ 7. (A) Eextracted ion chromatogram, (B) extracted ion mobilogram, and (C) detailed ion results.

The CCS values, a new parameter introduced in TIMS that is unique for every compound, further enhanced the confidence level of the identification of the compounds in addition to retention time and mass spectra. Based on the literature, to achieve an accurate identification of every investigated analyte, the %ΔCCS needs to be lower than 2%.^32^ Based on this, the CCS values of all the analytes that were identified in the RJ samples were measured both in standard solutions and in RJ samples, and the %ΔCCS values were calculated. All the %ΔCCS were lower than 2%, as shown in Table 3, and therefore considered acceptable as an additional identification feature of the method.

**Table 3:** %ΔCCS as Calculated Evaluating the CCS Matrix Effect

analyte	CCS (Å^2^) standard analysis	mean CCS (Å^2^) Royal Jelly analysis	%ΔCCS
2-*cis*,4-*trans*-abscisic acid	166.6	166.8	0.12
4-hydroxybenzaldehyde	116.4	116.6	0.17
4-hydroxybenzoic acid	118.7	119.5	0.67
acacetin	167.3	164.7	1.6
apigenin	157.6	157.7	0.063
benzoic acid	117.2	116.6	0.51
chrysin	156.2	156.2	0.0
eriodictyol	164.5	164.3	0.12
ethyl caffeate	146.8	147.1	0.20
ethyl gallate	138.7	138.6	0.072
ferulic acid	138.8	138.5	0.22
galangin	155.7	156.3	0.39
genistein	160.3	157.7	1.6
kaempferide	167.5	167.4	0.060
kaempferol	160.8	160.9	0.062
luteolin	160.4	160.6	0.12
naringenin	162.7	163.5	0.49
*p*-coumaric acid	129.1	129.4	0.23
pinobanksin	158.1	158.6	0.32
pinocembrin	157.8	158.2	0.25
quercetin	163.5	163.7	0.12
quinic acid	134.4	134.5	0.074
sakuranetin	169.1	168.1	0.59
syringaldehyde	137.8	136.4	1.0

The findings of the analysis of the 22 RJ samples are summarized in Table 4, where the minimum, maximum, and average concentrations of the bioactive compounds determined in the RJ samples, along with the %frequency of occurrence, are presented in alphabetical order. The detailed results of each sample bioactive profile are included in Table S10. Furthermore, the quantitative results of Table S10 are presented in the form of a heatmap in Figure S5, which visualizes the variation of each analyte within the samples.

**Table 4:** Findings of the Present Study, Minimum, Maximum, and Average Concentration (μg g^–1^) of Phenolic Compounds and Three Organic Acids in Royal Jelly Samples, Their Frequency of Detection, and the Relative Literature Findings

analyte	class	minimum concentration (μg g^–1^)	maximum concentration (μg g^–1^)	average concentration (μg g^–1^)	frequency of detection (%)	literature references
2-*cis*,4-*trans*-abscisic acid	organic acid	0.098	2.1	0.59	100	not reported
4-hydroxybenzaldehyde	phenolic aldehyde	0.12	1.7	0.58	100	not reported
4-hydroxybenzoic acid	phenolic acid	0.64	3.6	1.8	100	0.01–0.3% in RJ ether extract^64^
						2.910 mg g^–1^ ^47^
						2.489 mg g^–1^ in RJ extract^46^
acacetin	flavonoid	0.24	6.1	1.1	100	<LOD (0.005)–0.25 μg g^–1^ in RJ products^16^
apigenin	flavonoid	0.11	1.3	0.37	100	<LOD (0.005)–0.112 μg g^–1^ in RJ products^16^
benzoic acid	organic acid	0.13	1.6	0.58	100	3.08% TIC^a^ of RJ^65^
						2.8–3.6% of RJ volatile compounds^64^
chrysin	flavonoid	0.22	11	2.1	100	0.065 ± 0.005 μg g^–1^ in RJ^14^
						<LOD (0.020)–0.267 μg g^–1^ in RJ products^16^
eriodictyol	flavonoid	<LOQ (0.10)	0.52	0.17	100	not reported
ethyl caffeate	phenolic ester	<LOD (0.024)	0.47	0.15	91	not reported
ethyl gallate	phenolic ester	0.25	1.7	0.59	100	not reported
ferulic acid	phenolic acid	<LOD (0.031)	5.5	0.85	82	68.42–74.31 mg/100 mg in RJ protein^12^
						13.0–18.9 μg g^–1^ in RJ products^16^
						126.6–129.6 μg g^–1^ dry RJ^43^
galangin	flavonoid	0.22	2.4	0.69	100	0.51–0.57 mg/100 mg in RJ protein^12^
genistein	flavonoid	0.12	1.2	0.37	100	<LOD (0.020)–0.699 μg g^–1^ in RJ products^16^ 4 μg g^–1^ ^47^
kaempferide	flavonoid	0.15	1.3	0.45	100	not reported
kaempferol	flavonoid	0.54	4.8	1.6	100	0.294 ± 0.015 μg g^–1^ in RJ^14^
luteolin	flavonoid	0.33	4.5	1.0	100	5 μg g^–1^ ^47^
naringenin	flavonoid	<LOD (0.023)	0.95	0.18	95	0.041 ± 0.003 μg g^–1^ in RJ^14^
						<LOD (0.020)–0.793 μg g^–1^ in RJ products^16^
*p*-coumaric acid	phenolic acid	<LOD (0.043)	16	3.7	91	trace in RJ ether extract^64^
						39.6 μg g^–1^ dry RJ^43^
pinobanksin	flavonoid	<LOQ (0.068)	1.7	0.34	100	0.3% TIC^a^ of RJ^65^
pinocembrin	flavonoid	0.13	6.4	0.99	100	not reported
quercetin	flavonoid	0.68	10	2.8	100	15.96–18.44 mg/100 mg in RJ protein^12^
						0.205 ± 0.014 μg g^–1^ in RJ^14^
quinic acid	organic acid	<LOD (0.038)	18	3.8	91	trace −0.3% in RJ methanol extract^64^
						6.573 mg g^–1^ ^47^
						16.66 mg g^–1^ in RJ extract^46^
sakuranetin	flavonoid	<LOD (0.040)	15	2.2	95	<LOD (0.020)–0.692 μg g^–1^ in RJ products^16^
syringaldehyde	phenolic aldehyde	0.15	1.3	0.41	100	not reported

a TIC: Total ion current of the GC chromatogram.

It is noted that 24 compounds were identified and quantified with the new validated method. All the compounds were determined with more than 80% frequency of occurrence, while 18 of them were detected in all tested samples (100% frequency of occurrence), indicating a homogeneity with regard to the phenolic profile and the presence of 2-*cis*,4-*trans*-abscisic acid, benzoic acid, and quinic acid of the Greek RJ collected from different geographical regions of the country. However, a significant variation was noted among the samples’ concentrations.

Sixteen of these compounds have been previously reported in the literature in RJ or RJ products or extracts, while eight of them were determined and quantified for the first time in the present study. These compounds are 2-*cis*,4-*trans*-abscisic acid; 4-hydroxybenzaldehyde; eriodictyol; ethyl caffeate; ethyl gallate; kaempferide; pinocembrin; and syringaldehyde, and they are all known naturally occurring compounds in plants and bee products with bioactive properties.^14,16,64–66^

Regarding the concentration levels of the anaytes, the latest can be categorized into three groups based on their average concentration values. The first group with the highest average concentrations, ranging between 1.1 and 3.8 μg g^–1^ included in descending order: quinic acid > *p*-coumaric acid > quercetin > sakuranetin > chrysin > 4-hydroxybenzoic acid > kaempferol > acacetin. The second group with average concentrations between 0.58 and 1.0 μg g^–1^ included: luteolin > pinocembrin > ferulic acid > galangin > 2-*cis*,4-*trans*-abscisic acid > ethyl gallate >4-hydroxybenzaldehyde > benzoic acid. The third group with the lowest average concentrations, ranging between 0.15 and 0.45 μg g^–1^ included in descending order: kaempferide > syringaldehyde > apigenin > genistein > pinobanksin > naringenin > eriodictyol > ethyl caffeate.

Overall, the most abundant bioactive compound was quinic acid, with an average concentration of 3.8 μg g^–1^. The highest concentration of quinic acid was 18 μg g^–1^ and it was determined in a sample from Arcadia in Peloponesse harvested in the Summer of 2023 (RJ8) (Table S1). In two recent studies, quinic acid has been proposed as the dominant compound of RJ from Bingöl and Artvin, two regions of Turkey.^46,47^ Quinic acid has been identified again, in methanol extracts of RJ from Poland with GC-MS, at a trace level up to 0.3% relative composition of the methanol extracts.^64^ Although quinic acid is not a phenolic compound, but a polyol, it has been reported to possess antioxidant, anticancer, antimicrobial, antiviral, antidiabetic, and antiaging properties, and it is usually found in medicinal plants.^67^ Along with quinic acid, three other acids, benzoic acid,^64,65^ 4-hydroxybenzoic acid,^64^ and *p*-coumaric acid,^64^ as well as the flavonoid pinobanksin^65^ have also been reported to be part of RJ composition from Poland^64^ and Turkey^65^ after GC-MS analysis.

Comparison of the findings of the present study with results obtained from the literature was not an easy task, since many of the studies report their results in different extracts of RJ. Despite that, comparison of the concentrations reported in RJ or RJ products revealed that there were cases where the findings of the present study conducted in RJ originated from Greece had a common concentration range with other studies conducted in Spain, like the cases of genistein^16^ with a combined concentration range from both studies between <0.020 and 1.2 μg g^–1^, naringenin^14,16^ with combined concentration range from the three studies <0.020–0.95 μg g^–1^, and sakuranetin^16^ with a combined concentration range from both studies between <0.020 and 15 μg g^–1^. On the other hand, there were compounds like acacetin, apigenin, chrysin, and kaempferol found in the present study at higher concentrations in comparison to the Spanish studies^14,16^ as it can be observed in Table 4. On the contrary, the quantified concentrations of ferulic acid did not exceed 5.5 μg g^–1^ in Greek RJ, while in the Spanish study,^16^ its concentration ranged between 13.0 and 18.9 μg g^–1^ in RJ products and in a Romanian study^43^ from 126.6 to 129.6 μg g^–1^ of dry weight RJ. These variations could possibly be attributed to the different geographical origin of the raw material of RJ. Galangin, one more flavonoid determined in the present study, at a mean concentration of 0.69 μg g^–1^, was also determined in RJ protein originating from Taiwan.^12^ In the present study, the concentration of luteolin ranged from 0.33 to 4.5 μg g^–1^. Luteolin has been reported in a Turkish study containing only one sample at a concentration of 5 μg g^–1^.^47^

Many of the natural compounds determined in this study exhibit bioactivities that are relevant to their health-promoting properties. It is noteworthy that quercetin, a compound found in all the tested samples at concentrations up to 10 μg g^–1^, has been used in alzheimer and asthma treatment, and it is known for its antiobesity, antihypertensive, and antiallergic activities.^68^ The new compounds demonstrated diverse beneficial bioactivities. Specifically, ethyl gallate and eriodictyol both show significant anticancer effects through modulation of important signaling pathways and apoptosis.^69–71^ Ethyl caffeate also presents anticancer activity against human ovarian and breast cancer. In addition, it exhibits antiviral properties against HIV-1 and HBV.^72^ Another polyphenol, kaempferide, possesses multiple beneficial biological functions, including protecting from osteoporosis and reducing oxidative stress.^73^ In addition, syringaldehyde and 4-hydroxybenzaldehyde show antimicrobial and gut-regulating effects.^74,75^ The compound 2-*cis*,4-*trans* abscisic acid reduces glycemia without increasing insulinemia, improves lipidemia, and lowers cardiovascular risk.^76^ Furthermore, pinocembrin, another compound that lowers cardiovascular risk, appears promising against inflammatory disorders and ischemic stroke.^77^ The beneficial properties of the determined compounds in the Greek RJ of the present study are summarized in Table S11, and they include antimicrobial, anticancer, anti-inflammatory, antifungal, antioxidant, antibacterial, antiallergic, antidiabetic, and neuroprotective properties.

To summarize, a cutting-edge UHPLC-TIMS-QTOF-MS method was developed with VIP-HESI as the ionization source for the determination of 61 bioactive compounds, mainly polyphenols in RJ. Following an optimization approach, it was found that capillary voltage, probe gas temperature, and probe gas flow were the critical parameters for the method’s performance. The optimum values included: sprayer’s position; Near Up, nebulizer gas pressure, 3.5 bar; capillary voltage, 2500 V; dry gas flow, 10 L min^–1^; dry gas temperature, 230 °C; probe gas flow, 5 L min^–1^; and probe gas temperature, 450 °C. This combination provided increased sensitivity and facilitated the determination of analytes with different chemical structures, retention times, and *m*/*z*. The optimized protocol was then validated regarding linearity, LODs, LOQs, matrix effect, trueness, and precision, and satisfactory results were achieved for all of the evaluated parameters. Following target screening in 22 RJ samples collected across Greece, 24 compounds were identified and quantified, eight of them for the first time (2-*cis*,4-*trans*-abscisic acid; 4-hydroxybenzaldehyde; eriodictyol; ethyl caffeate; ethyl gallate; kaempferide; pinocembrin and syringaldehyde), verifying the applicability and success of the developed method. In conclusion, this target screening methodology resulted in a wide qualitative and quantitative chemical characterization of Greek RJ’s bioactive profile, providing additional information to the existing literature. Furthermore, the proposed analytical method could be applied in authentication studies of RJ and for the determination of relevant geographical and other chemical indicators. This could be a useful tool to detect fraud and safeguard the fair trade of this valuable functional food.

## Supplementary Material



## Notes

Geographical origin and harvest period of royal jelly samples harvested in 2023 (Table S1); Plackett–Burman design of variables in coded levels (Table S2); Box–Behnken design of variables in coded levels (Table S3); effects and regression coefficients of the analytes in Plackett–Burman design (Table S4); sum of squares and corresponding *F* and *p* values for the analytes in six variable Plackett–Burman design (Table S5); regression equations and *R* ^2^, adjusted *R* ^2^, and predicted *R* ^2^ values for six analytes (Table S6); ANOVA results of Box–Behnken Design for apigenin, chrysin, pinobanksin, quercetin, scopoletin and 2-*cis*,4-*trans* abscisic acid (Table S7); UHPLC-VIP-HESI-TIMS-QTOF-MS method performance parameters in terms of correlation coefficient, LODs, LOQs, matrix effect, and recovery evaluated at 3 concentration levels, low level (0.25 μg g^–1^), medium level (1 μg g^–1^), and high level (5 μg g^–1^) (Table S8); repeatability and intermediate precision of UHPLC-VIP-HESI-TIMS-QTOF-MS method evaluated at 3 concentration levels, low level (0.25 μg g^–1^), medium level (1 μg g^–1^), and high level (5 μg g^–1^) (Table S9); concentration (μg g^–1^) of bioactive compounds in RJ samples (Table S10); health benefits of bioactive compounds found in RJ samples (Table S11); different positions of the VIP-HESI probe on the *y*-axis (Figure S1); bar charts of the area of chromatograms as a function of nebulizer gas pressure (bars) in different sprayer positions for five target analytes (Figure S2); response surface plots between 1: capillary voltage and probe gas flow and 2: capillary voltage and probe gas temperature for A: Apigenin, B: Pinobanksin, C: Quercetin, D: Scopoletin, and E: 2-*cis*,4-*trans* abscisic acid (Figure S3); contour plots showing the analytes’ desirability between A: Capillary voltage and Probe gas flow, and B: Capillary voltage and Probe gas temperature (Figure S4); variation of the concentration of each analyte within RJ samples derived from the quantification results (Figure S5) (PDF)

## Abbreviations used

1/*k*_0_: inverse ion mobility
Å^2^: square angströms
ANOVA: analysis of variance
CCS: collision cross-section
EIC: extracted ion chromatogram
EIM: extracted ion mobilogram
ESI: electrospray ionization
GC: gas chromatography
HESI: heated electrospray ionization
HPLC: high-performance liquid chromatography
HRMS: high-resolution mass spectrometry
IMS: ion mobility spectrometry
*k*_0_: reduced mobility
LC: liquid chromatography
LC-MS: liquid chromatography- mass spectrometry
LOD: limit of detection
LOQ: limit of quantification
*m*/*z*: mass-to-charge ratio
MeOH: methanol
MS: mass spectrometry
°C: degrees celsius
bbCID: broadband collision-induced dissociation
pH: potential of Hydrogen
QTOF: quadrupole time-of-flight
*R* ^2^: regression coefficient
RC: regenerated cellulose
RJ: Royal Jelly
RSD: relative standard deviation
RT: retention time
S/N: signal-to-noise ratio
TIC: total ion current
TIMS: trapped ion mobility spectrometry
TOF: time-of-flight
UHPLC: ultrahigh-performance liquid chromatography
UHPLC-VIP-HESI-TIMS-QTOF-MS: ultrahigh-performance liquid chromatography-vacuum-insulated probe-heated electrospray ionization-trapped ion mobility spectrometry-quadrupole-time-of-flight mass spectrometry
UV: ultraviolet
VIP: vacuum-insulated probe
VIP-HESI: vacuum-insulated probe-heated electrospray ionization
