# Supplementary material for: Mapping Royal Jelly’s Bioactive Profile by Liquid Chromatography Coupled to Trapped Ion Mobility Spectrometry and High-Resolution Mass Spectrometry
Source: J Agric Food Chem. 2025 Sep 8;73(37):23684–99. doi: 10.1021/acs.jafc.5c04904 (PMC12447487; doi:10.1021/acs.jafc.5c04904)
Supplement: Supplementary file 1 [file jf5c04904_si_001.pdf]

SUPPLEMENTARY MATERIAL FOR

Mapping Royal Jelly's Bioactive Profile by Liquid Chromatography coupled to Trapped Ion Mobility Spectrometry and High Resolution Mass Spectrometry

Eleni S. Nastou[a], Dafni A. Preza-Mayo-Kataki[a], Panagiotis-Loukas P. Gialouris[a], Niki C. Maragou[b,a], Evagelos E. Gikas[a], Nikolaos S. Thomaidis[a *]

[a] Laboratory of Analytical Chemistry, Department of Chemistry, National and Kapodistrian University of Athens, Panepistimioupolis Zografou, 15771 Athens, Greece

[b] Department of Wine, Vine & Beverage Sciences, University of West Attica, 28 Ag. Spyridonos str, 12243 Egaleo, Greece

Table S1: Geographical origin and harvest period of royal jelly samples harvested in 2023.

Code	Administrative region	Exact area	Harvest Period
RJ 1	Peloponesse	Argolis	Spring
RJ 2	Western Greece	Elis	Spring
RJ 3	Western Greece	Elis	Summer
RJ 4	Epirus	Arta	Summer
RJ 5	Peloponnese	Laconia	Spring
RJ 6	Central Greece	Evoia	Spring
RJ 7	Western Macedonia	Florina	Summer
RJ 8	Peloponnese	Arcadia	Summer
RJ 9	Peloponnese	Argolis	Spring
RJ 10	Athos	Chalkidiki	Spring
RJ 11	Athos	Chalkidiki	Summer
RJ 12	Central Greece	Evoia	Summer
RJ 13	Epirus	Arta	Spring
RJ 14	Peloponnese	Laconia	Summer
RJ 15	Peloponesse	Argolis	Spring
RJ 16	Athos	Chalkidiki	Summer
RJ 17	Western Macedonia	Florina	Summer
RJ 18	Western Greece	Elis	Spring
RJ 19	Athos	Chalkidiki	Summer
RJ 20	Peloponnese	Arcadia	Spring
RJ 21	Western Greece	Elis	Summer
RJ 22	Peloponnese	Argolis	Spring

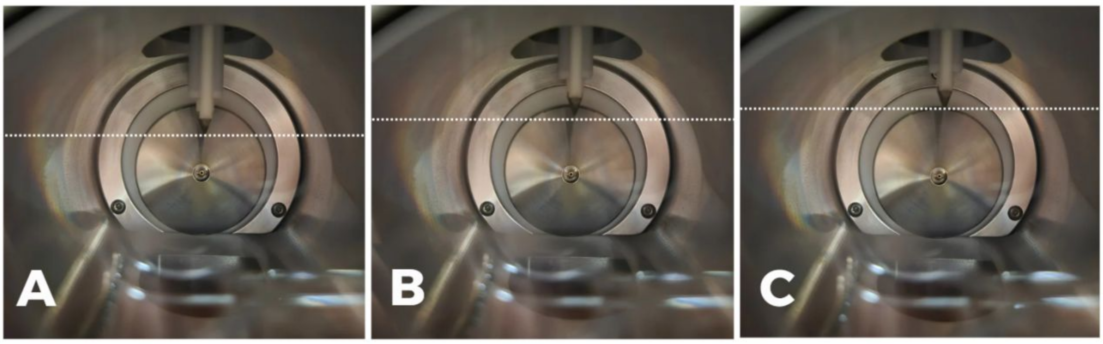

Figure S1: The different positions of the VIP-HESI probe on the y-axis. A: position Down (-4mm), B: position Middle (0mm), C: position Up (+4mm)

Table S2: Plackett-Burman design of variables in coded levels.

Run	A: Capillary Voltage (V)	B: Dry gas flow rate (L min^{-1})	C: Dry gas temperature(°C)	D: Probe gas flow rate (L min^{-1})	E: Probe gas temperature(°C)	F	G	H	J	K	L
1	-1	1	-1	1	1	-1	1	1	1	-1	-1
2	-1	-1	-1	1	-1	1	1	-1	1	1	1
3	1	-1	1	1	1	-1	-1	-1	1	-1	1
4	0	0	0	0	0	0	0	0	0	0	0
5	1	1	-1	-1	-1	1	-1	1	1	-1	1
6	1	-1	-1	-1	1	-1	1	1	-1	1	1
7	0	0	0	0	0	0	0	0	0	0	0
8	-1	1	1	1	-1	-1	-1	1	-1	1	1
9	1	-1	1	1	-1	1	1	1	-1	-1	-1
10	0	0	0	0	0	0	0	0	0	0	0
11	1	1	1	-1	-1	-1	1	-1	1	1	-1
12	-1	-1	-1	-1	-1	-1	-1	-1	-1	-1	-1
13	1	1	-1	1	1	1	-1	-1	-1	1	-1
14	-1	-1	1	-1	1	1	-1	1	1	1	-1
15	-1	1	1	-1	1	1	1	-1	-1	-1	1
16	0	0	0	0	0	0	0	0	0	0	0

Table S3: Box-Behnken design of variables in coded levels.

Run	A: Capillary Voltage (V)	B: Probe gas flow rate (L min^{-1})	C: Probe gas temperature (°C)
1	-1	0	1
2	1	-1	0
3	-1	-1	0
4	1	1	0
5	0	-1	-1
6	0	0	0
7	1	0	-1
8	1	0	1
9	0	1	-1
10	0	0	0
11	-1	0	-1
12	0	-1	1
13	0	1	1
14	0	0	0
15	0	0	0
16	-1	1	0

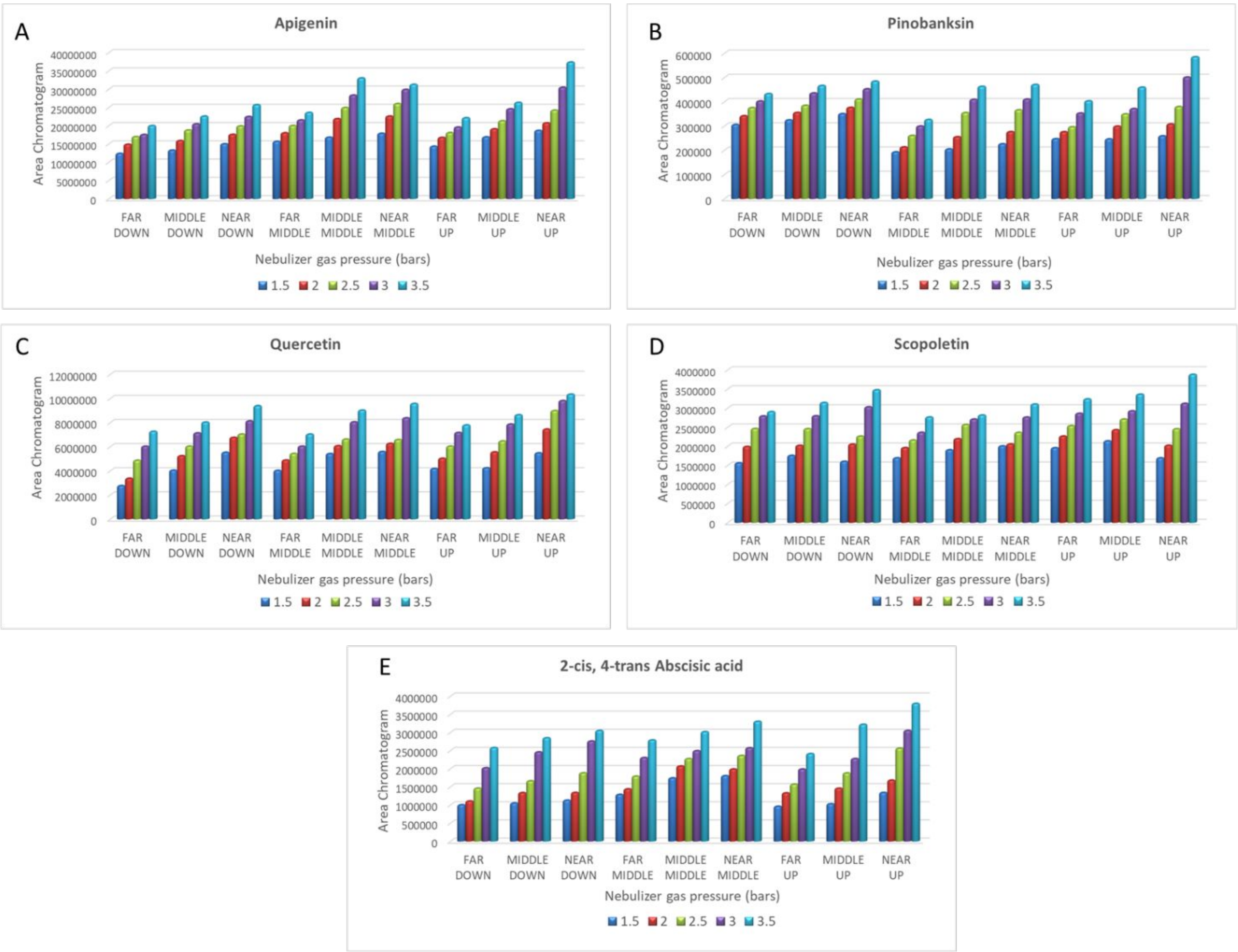

Figure S2: Bar charts of the area of chromatograms as a function of nebulizer gas pressure (bars) in different sprayer positions for five target analytes (A: Apigenin, B: Pinobanksin, C: Quercetin, D: Scopoletin, E: 2-cis,4-trans Abscisic acid)

Table S4: Effects and regression coefficients of the analytes in Plackett-Burman Design.

	Effect	Regression Coefficient	Standard Error
		Apigenin	
Intercept		17.33	0.0143
A: Capillary Voltage	-1.2786	-0.6393	0.0143
B: Dry Gas Flow	0.0063	0.0032	0.0143
C: Dry Gas Temperature	-0.0609	-0.0305	0.0143
D: Probe gas flow	0.1688	0.0844	0.0143
E: Probe gas temperature	0.3487	0.1744	0.0143
		Chrysin	
Intercept		18.30	0.0113
A: Capillary Voltage	-1.0094	-0.5047	0.0113
B: Dry Gas Flow	0.0008	0.0042	0.0113
C: Dry Gas Temperature	-0.0181	-0.0091	0.0113
D: Probe gas flow	0.1427	0.0714	0.0113
E: Probe gas temperature	0.2548	0.1274	0.0113
		Pinobanksin	
Intercept		12.80	0.0203
A: Capillary Voltage	-1.2353	-0.6177	0.0203
B: Dry Gas Flow	0.0512	0.0256	0.0203
C: Dry Gas Temperature	-0.0342	-0.0171	0.0203
D: Probe gas flow	0.2085	0.1043	0.0203
E: Probe gas temperature	0.3494	0.1747	0.0203
		Quercetin	
Intercept		2698	24.92
A: Capillary Voltage	-2040	-1020	24.92
B: Dry Gas Flow	82.87	41.44	24.92
C: Dry Gas Temperature	-39.48	-19.74	24.92
D: Probe gas flow	182.97	91.48	24.92
E: Probe gas temperature	346.09	173.0	24.92
		Scopoletin	
Intercept		14.35	0.0256
A: Capillary Voltage	-1.365	-0.6827	0.0256
B: Dry Gas Flow	0.1021	0.0511	0.0256
C: Dry Gas Temperature	-0.0555	-0.0278	0.0256
D: Probe gas flow	0.1698	0.0849	0.0256
E: Probe gas temperature	0.3556	0.1778	0.0256
		2cis,4-trans Abscisic acid	
Intercept		15.05	0.0144
A: Capillary Voltage	-1.310	-0.6551	0.0144
B: Dry Gas Flow	-0.0151	-0.0075	0.0144
C: Dry Gas Temperature	-0.0307	-0.0153	0.0144
D: Probe gas flow	0.1845	0.0923	0.0144
E: Probe gas temperature	0.3281	0.1641	0.0144

Table S5: Sum of squares and corresponding F and p values for the analytes in six variable Plackett–Burman design.

	Sum of squares	df	Mean Square	F-Value	p-Value
			Apigenin		
Model	5.37	5	1.07	434.9	< 0.0001
A: Capillary Voltage	4.90	1	4.90	1987	< 0.0001
B: Dry Gas Flow	0.0001	1	0.0001	0.0485	0.8306
C: Dry Gas Temperature	0.0111	1	0.0111	4.51	0.0626
D: Probe gas flow	0.0855	1	0.0855	34.66	0.0002
E: Probe gas temperature	0.3650	1	0.3650	147.89	< 0.0001
			Chrysin		
Model	3.31	5	0.6628	435.9	< 0.0001
A: Capillary Voltage	3.06	1	3.06	2010	< 0.0001
B: Dry Gas Flow	0.0002	1	0.0002	0.1363	0.7205
C: Dry Gas Temperature	0.0010	1	0.0010	0.6471	0.4419
D: Probe gas flow	0.0611	1	0.0611	40.21	0.0001
E: Probe gas temperature	0.1948	1	0.1948	128.1	< 0.0001
			Pinobanksin		
Model	5.09	5	1.02	206.5	< 0.0001
A: Capillary Voltage	4.58	1	4.58	929.5	< 0.0001
B: Dry Gas Flow	0.0079	1	0.0079	1.60	0.2380
C: Dry Gas Temperature	0.0035	1	0.0035	0.7110	0.4210
D: Probe gas flow	0.1305	1	0.1305	26.49	0.0006
E: Probe gas temperature	0.3661	1	0.3661	74.34	< 0.0001
			Quercetin		
Model	1.298E+07	5	2.595E+06	348.4	< 0.0001
A: Capillary Voltage	1.249E+07	1	1.249E+07	1676	< 0.0001
B: Dry Gas Flow	20602	1	20602.64	2.77	0.1307
C: Dry Gas Temperature	4676	1	4677	0.6278	0.4485
D: Probe gas flow	1.004E+05	1	1.004E+05	13.48	0.0051
E: Probe gas temperature	3.593E+05	1	3.593E+05	48.24	< 0.0001
			Scopoletin		
Model	6.10	5	1.22	154.8	< 0.0001
A: Capillary Voltage	5.59	1	5.59	709.6	< 0.0001
B: Dry Gas Flow	0.0313	1	0.0313	3.97	0.0775
C: Dry Gas Temperature	0.0093	1	0.0093	1.17	0.3068
D: Probe gas flow	0.0865	1	0.0865	10.97	0.0091
E: Probe gas temperature	0.3794	1	0.3794	48.14	< 0.0001
			2cis,4-trans Abscisic acid		
Model	5.58	5	1.12	449.6	< 0.0001
A: Capillary Voltage	5.15	1	5.15	2075	< 0.0001
B: Dry Gas Flow	0.0007	1	0.0007	0.2752	0.6125
C: Dry Gas Temperature	0.0028	1	0.0028	1.14	0.3136
D: Probe gas flow	0.1022	1	0.1022	41.19	0.0001
E: Probe gas temperature	0.3231	1	0.3231	130.2	< 0.0001

Table S6: The regression equations and R^2, adjusted R^2, and predicted R^2 values for six analytes.

Analytes	Equation	R^2	Adjusted R^2	Predicted R^2
Apigenin	Y=17.33-0.6393A+0.0320B-0.0305C+0.0844D+0.1744E	0.996	0.994	0.98
Chrysin	Y=18.30-0.5047A+0.0042B-0.0091C+0.0714D+0.1274E	0.996	0.994	0.98
Pinobanksin	Y=12.80-0.6170A+0.0256B-0.0170C+0.1043D+0.1740E	0.991	0.990	0.98
Quercetin	Y=2698-1020.29A+41.44B-19.74C+91.48D+173.0E	0.995	0.992	0.98
Scopoletin	Y=14.35-0.6827A+0.0511B-0.0278C+0.0849D+0.1778E	0.98	0.977	0.96
2-cis,4-trans Absisic acid	Y=15.05-0.6551A-0.0075B-0.0153C+0.0923D+0.1641E	0.996	0.994	0.98

Table S7: ANOVA analysis results of Box–Behnken Design for apigenin, chrysin, pinobanksin, quercetin, scopoletin, and 2-cis,4-trans abscisic acid.

	F-Value	p-Value	Equations	R^2	Adjusted R^2	Predicted R^2
			Apigenin			
Model	212.6	< 0.0001	Y= 6148.3			
A: Capillary Voltage	1732	< 0.0001	-1334.87A			
B: Probe Gas Flow	24.94	0.0025	+147.68B			
C: Probe Gas Temperature	67.42	0.0002	+250.00C			
AB	3.59	0.1068	-49.87AB	0.997	0.992	0.95
AC	3.59	0.1068	-16.36AC			
BC	0.8109	0.4025	+34.88BC			
A^2	70.77	0.0002	+134.75A^2			
B^2	4.25	0.0849	-99.26B^2			
C^2	5.07	0.0653	-94.72C^2			
			Chrysin			
Model	43.93	< 0.0001	Y= 0.0001			
A: Capillary Voltage	325.0	< 0.0001	-2.06e-05A			
B: Probe Gas Flow	6.96	0.0386	-2.085e-06B			
C: Probe Gas Temperature	21.9	0.0034	+4.33e-06C			
AB	4.20	0.0864	+1.516e-06AB			
AC	9.40	0.0220	-9.342e-07AC	0.990	0.96	0.96
BC	0.0512	0.8286	-2.456e-07BC			
A^2	27.3	0.0020	+6.312e-07A^2			
B^2	0.3638	0.5685	1.373e-06B^2			
C^2	0.1720	0.6928	4.150e-07C^2			
			Pinobanksin			
Model	243.9	< 0.0001	Y= 2.32e-06			
A: Capillary Voltage	1952	< 0.0001	+1.25e-06A			
B: Probe Gas Flow	46.88	0.0005	-1.47e-07B			
C: Probe Gas Temperature	171.6	< 0.0001	-3.09e-07C			
AB	3.45	0.1126	-3.32e-08AB	0.997	0.993	0.96
AC	9.22	0.0229	-1.435e-07AC			
BC	2.31	0.1797	- 5.06e-10BC			
A^2	0.0051	0.9453	+5.99e-07A^2			
B^2	7.14	0.0369	+9.83e-08B^2			
C^2	2.47	0.1672	+7.70e-08C^2			
			Quercetin			
Model	249.4	< 0.0001	Y= 0.0004			
A: Capillary Voltage	2054	< 0.0001	+0.0001A			
B: Probe Gas Flow	22.94	0.0030	-9.61e-06B			
C: Probe Gas Temperature	125.9	< 0.0001	-2.42e-05C			
AB	0.7067	0.4328	+3.62e-06AB	0.997	0.993	0.96
AC	0.0278	0.8732	-1.53e-06AC			
BC	0.0087	0.9288	-2.94e-07BC			
A^2	23.56	0.0028	+4.36e-05A^2			
B^2	8.93	0.0244	+6.39e-06B^2			
C^2	8.65	0.0259	+1.16e-05C^2			
			Scopoletin			
Model	1676	< 0.0001	Y= 1236			
A: Capillary Voltage	13310	< 0.0001	-412.43A			
B: Probe Gas Flow	217.0	< 0.0001	+48.59B			
C: Probe Gas Temperature	893.9	< 0.0001	+105.55C			
AB	55.04	0.0003	-34.13AB	0.997	0.9990	0.997
AC	78.32	0.0001	-29.74AC			
BC	1.83	0.2251	+5.91BC			
A^2	424.2	< 0.0001	+68.27 A^2			
B^2	30.04	0.0015	-23.33B^2			
C^2	76.10	0.0001	-41.14C^2			
			2cis,4-trans Abscisic acid			
Model	211.0	< 0.0001	Y= 1802.36			
A: Capillary Voltage	1755	< 0.0001	-469.31A			
B: Probe Gas Flow	21.8	0.0034	+49.47B			
C: Probe Gas Temperature	82.8	< 0.0001	+92.69C			
AB	0.0114	0.9183	-25.05AB	0.997	0.992	0.96
AC	1.49	0.2684	-34.05AC			
BC	3.50	0.1107	+26.92BC			
A^2	1.13	0.3284	+36.45A^2			
B^2	14.1	0.0094	-63.98B^2			
C^2	18.7	0.0050	-43.30C^2			

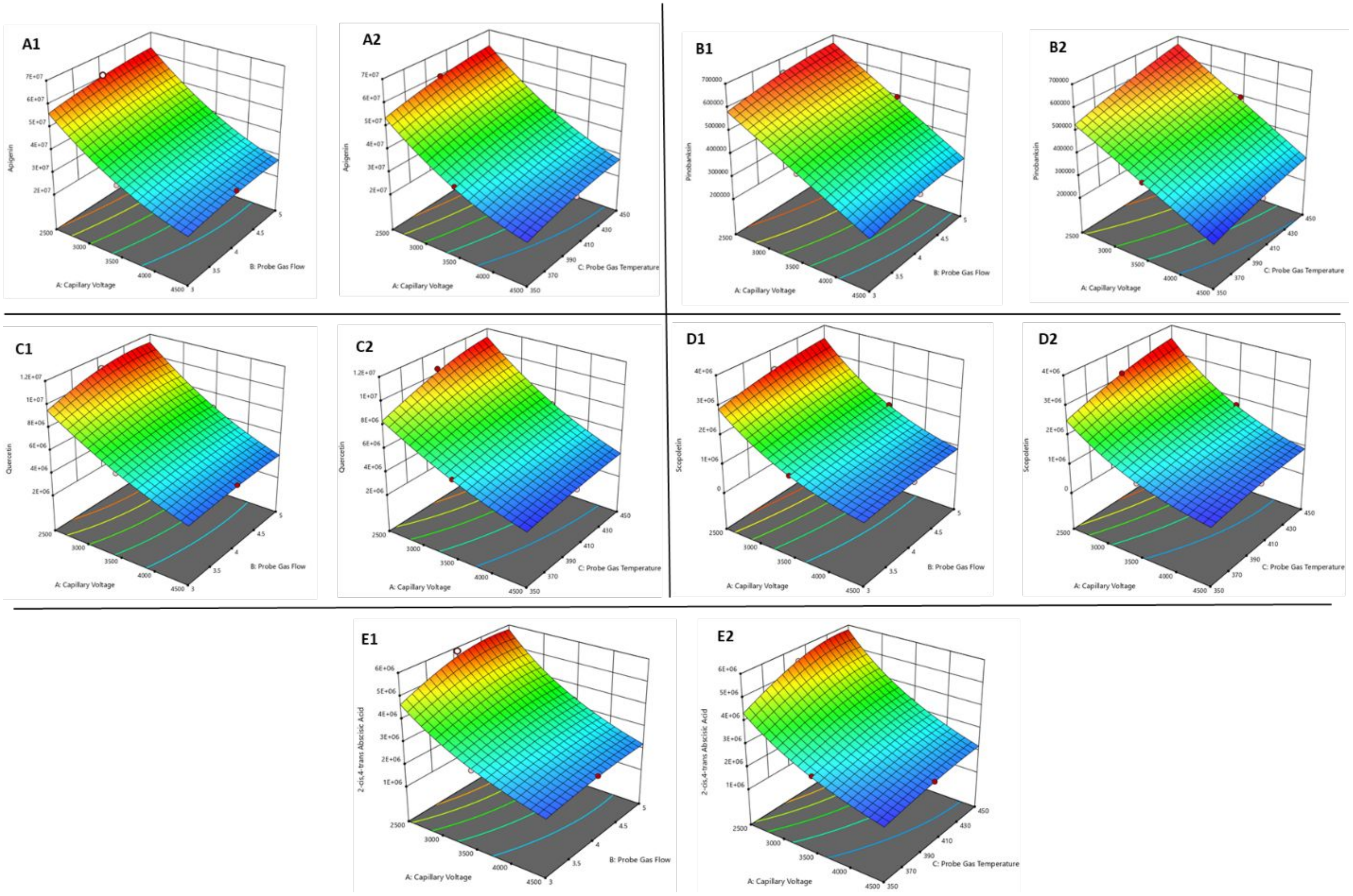

Figure S3: Response surface plots between 1: capillary voltage and probe gas flow, and 2: capillary voltage and probe gas temperature for A: Apigenin, B: Pinobanksin, C: Quercetin, D: Scopoletin, and E: 2-cis,4-trans Abscisic acid.

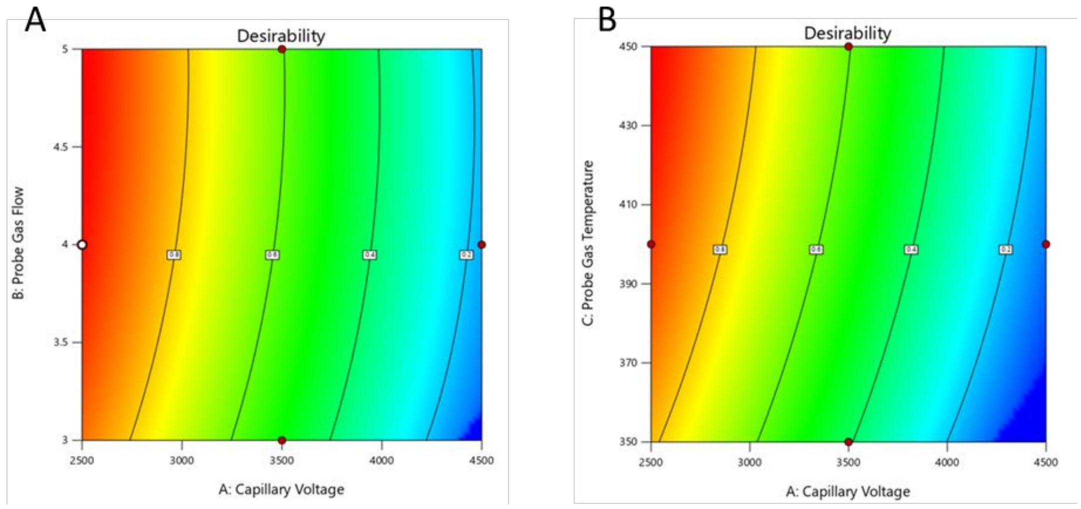

Figure S4: Contour plots showing the analytes' desirability between A: Capillary voltage and Probe gas flow, and B: Capillary voltage and Probe gas temperature.

Table S8: UHPLC-VIP-HESI-TIMS-QTOF-MS method performance parameters in terms of correlation coefficient, LODs, LOQs, Matrix Effect and recovery evaluated at 3 concentration levels, low level (0.25 µg g^{-1}), medium level (1 µg g^{-1}) and high level (5 µg g^{-1}).

Analyte	R^2	LOD (µg g^{-1})	LOQ (µg g^{-1})	Matrix Effect%	Recovery% Low Level	Recovery% Medium Level	Recovery% High Level
2-cis,4-trans-Abscisic acid	0.994	0.0013	0.0044	-9	105	99	95
3,4-Dihydroxybenzoic acid (Protocatechuic acid)	0.991	0.039	0.13	38	87	91	91
4-Hydroxybenzaldehyde	0.990	0.014	0.048	-91	84	96	97
4-Hydroxybenzoic acid	0.993	0.012	0.041	100	92	97	95
Acacetin	0.98	0.0065	0.022	-20	99	108	96
Apigenin	0.98	0.016	0.055	-22	94	103	95
Benzoic acid	0.996	0.037	0.12	-155	97	100	94
Caffeic acid	0.998	0.069	0.23	50	92	93	94
Catechin	0.9990	0.076	0.25	15	98	98	93
Catechol	0.995	0.088	0.29	-118	91	95	99
Chlorogenic acid	0.994	0.024	0.079	40	95	95	99
Chrysin	0.992	0.0035	0.012	-3	84	95	94
Diosmetin	0.98	0.075	0.25	2	86	94	101
Epicatechin	0.997	0.035	0.12	19	86	89	106
Eriodictyol	0.990	0.031	0.10	-34	80	91	93
Ethyl caffeate	0.98	0.024	0.080	-41	93	96	102
Ethyl gallate	0.990	0.074	0.25	-80	87	92	104
Ethyl vanillin	0.991	0.075	0.25	-63	92	99	94
Ferulic acid	0.995	0.031	0.10	-56	91	99	94
Fraxetin	0.991	0.085	0.28	-48	100	102	103
Fraxidin	0.993	0.052	0.17	24	82	95	98
Fraxin	0.993	0.0016	0.0052	-25	93	94	101
Galangin	0.994	0.066	0.22	-44	83	92	95
Gallic acid	0.9990	0.075	0.25	85	99	93	92
Genistein	0.990	0.013	0.044	-21	85	101	96
Hesperetin	0.991	0.0078	0.026	-33	90	109	102
Hydroxytyrosol	0.992	0.051	0.17	-95	84	87	99
Kaempferide	0.992	0.052	0.17	-50	92	100	98
Kaempferol	0.990	0.043	0.14	-37	87	94	104

Analyte	R^2	LOD (μg g^{-1})	LOQ (μg g^{-1})	Matrix Effect%	Recovery% Low Level	Recovery% Medium Level	Recovery% High Level
Luteolin	0.997	0.045	0.15	17	89	90	100
Maslinic acid	0.993	0.021	0.069	-62	89	88	98
Myricetin	0.994	0.024	0.08	-87	95	100	99
Naringenin	0.990	0.023	0.075	24	90	94	96
Naringin	0.998	0.014	0.045	6	89	92	97
p-Coumaric acid	0.998	0.043	0.14	-15	93	91	107
Phloretin	0.990	0.0029	0.0096	-16	95	101	97
Phloridzin	0.9990	0.0013	0.0042	27	91	93	100
Pinobanksin	0.991	0.020	0.068	28	91	95	98
Pinocembrin	0.993	0.0022	0.0072	-33	88	101	97
Polydatin	0.993	0.0031	0.010	18	93	91	99
Protocatechuic acid ethyl ester	0.995	0.047	0.16	-31	82	101	101
Quercetin	0.993	0.069	0.23	-48	93	94	102
Quinic acid	0.993	0.038	0.13	71	89	98	96
Resveratrol	0.992	0.040	0.13	60	94	93	103
Rosmarinic acid	0.991	0.012	0.040	36	87	96	101
Rutin	0.998	0.0029	0.0097	-22	96	92	104
Sakuranetin	0.994	0.040	0.13	-34	87	91	96
Salicylic acid	0.992	0.090	0.30	-22	82	88	93
Scopoletin	0.996	0.075	0.25	34	90	91	100
Sinapic acid	0.997	0.043	0.14	-52	94	97	100
Syringaldehyde	0.98	0.032	0.11	-27	95	95	99
Syringic acid	0.994	0.068	0.23	74	87	102	92
Tyrosol	0.992	0.058	0.19	-97	102	99	96
Vanillic acid	0.9990	0.084	0.28	46	93	106	96
Vanillin	0.995	0.075	0.25	-37	93	95	98

Table S9: Repeatability and intermediate precision of UHPLC-VIP-HESI-TIMS-QTOF-MS method evaluated at 3 concentration levels, low level (0.25 μg g^{-1}), medium level (1 μg g^{-1}), and high level (5 μg g^{-1}).

Analyte	RSD% (Low Level) Repeatability	RSD% (Medium Level) Repeatability	RSD% (High Level) Repeatability	RSD% (Low Level) Intermediate Precision	RSD% (Medium Level) Intermediate Precision	RSD% (High Level) Intermediate Precision
2-cis,4-trans-Abscisic acid	3.3	4.0	2.5	4.6	3.3	2.9
3,4-Dihydroxybenzoic acid (Protocatechuic acid)	3.1	3.0	3.5	3.3	4.7	3.9
4-Hydroxybenzaldehyde	2.8	6.6	4.2	3.7	6.3	5.3
4-Hydroxybenzoic acid	1.6	4.2	2.8	2.9	5.3	6.2
Acacetin	3.0	4.5	1.7	4.0	7.6	4.5
Apigenin	2.0	3.6	2.8	2.1	5.0	3.5
Benzoic acid	1.7	3.1	2.2	4.0	4.6	5.6
Caffeic acid	2.5	1.6	2.7	1.8	3.5	4.0
Catechin	4.1	2.0	2.1	4.5	3.1	2.6
Catechol	2.1	2.0	3.9	4.5	2.2	7.3
Chlorogenic acid	5.1	4.0	2.6	5.7	5.0	6.6
Chrysin	4.9	7.4	2.0	5.4	11	12
Diosmetin	1.8	2.1	2.8	6.8	2.5	6.8
Epicatechin	2.2	2.5	2.7	3.0	5.2	6.3
Eriodictyol	3.6	2.8	3.0	5.0	3.1	9.4
Ethyl caffeate	1.8	3.7	2.2	2.0	5.3	3.1
Ethyl gallate	2.1	1.4	4.2	2.5	1.6	4.3
Ethyl vanillin	3.3	2.5	1.9	5.0	3.6	2.5
Ferulic acid	5.3	2.0	2.6	5.7	3.0	7.0
Fraxetin	2.0	3.7	2.6	2.5	4.7	4.6
Fraxidin	3.2	5.7	4.8	4.3	7.5	6.8
Fraxin	2.1	2.5	1.8	2.8	3.6	2.6
Galangin	3.1	2.2	3.8	3.9	3.1	4.5
Gallic acid	2.0	3.5	2.2	5.6	4.1	2.9
Genistein	2.0	2.1	1.2	2.8	5.5	3.6
Hesperetin	2.8	3.5	2.0	4.1	5.1	4.6
Hydroxytyrosol	3.2	1.4	2.8	5.5	2.4	18

Analyte	RSD% (Low Level) Repeatability	RSD% (Medium Level) Repeatability	RSD% (High Level) Repeatability	RSD% (Low Level) Intermediate Precision	RSD% (Medium Level) Intermediate Precision	RSD% (High Level) Intermediate Precision
Kaempferide	3.5	2.2	2.0	4.4	5.7	3.7
Kaempferol	1.7	2.6	3.5	2.4	3.7	4.8
Luteolin	3.5	2.5	1.9	5.8	4.2	3.6
Maslinic acid	2.5	3.0	2.2	5.5	4.6	4.2
Myricetin	3.1	4.4	1.6	6.5	4.8	4.5
Naringenin	4.0	3.7	2.9	4.5	3.9	2.7
Naringin	4.3	3.1	2.7	5.4	3.5	5.7
p-Coumaric acid	5.4	1.1	1.4	7.6	1.3	3.0
Phloretin	1.6	2.4	5.5	2.6	3.5	4.6
Phloridzin	4.1	0.82	2.4	5.7	6.0	4.2
Pinobanksin	4.1	3.6	2.4	6.2	5.0	4.3
Pinocembrin	3.2	2.5	3.1	4.0	5.8	3.2
Polydatin	1.9	3.4	2.7	5.1	4.1	4.5
Protocatechuic acid ethyl ester	3.1	3.5	3.0	7.5	3.9	4.6
Quercetin	1.4	2.2	2.0	2.7	3.8	5.5
Quinic acid	2.6	1.6	3.7	6.3	4.7	4.6
Resveratrol	1.8	2.4	3.1	2.4	2.4	5.3
Rosmarinic acid	5.3	2.4	2.5	4.7	4.5	7.7
Rutin	1.3	3.7	2.4	4.8	4.5	3.6
Sakuranetin	3.5	2.4	1.5	5.5	4.1	2.3
Salicylic acid	4.6	3.7	2.4	6.7	5.4	4.0
Scopoletin	3.8	3.1	2.1	5.7	4.0	3.3
Sinapic acid	3.9	1.5	1.1	4.1	1.2	1.8
Syringaldehyde	2.0	4.4	2.8	2.5	6.1	5.9
Syringic acid	1.9	1.6	1.8	10	2.1	5.1
Tyrosol	3.4	2.7	3.1	3.9	7.0	5.9
Vanillic acid	4.9	6.5	3.0	6.0	7.4	5.6
Vanillin	2.8	3.4	1.6	4.3	3.7	3.3

Table S10: Concentration (μg g^{-1}) of bioactive compounds in RJ samples.

Analytes	RJ1	RJ2	RJ3	RJ4	RJ5	RJ6	RJ7	RJ8	RJ9	RJ10	RJ11	RJ12	RJ13	RJ14	RJ15	RJ16	RJ17	RJ18	RJ19	RJ20	RJ21	RJ22
2-cis,4-trans-Abscisic acid	0.23	0.32	1.1	0.52	2.1	0.62	0.62	0.39	0.74	0.67	0.13	0.26	0.46	1.7	0.63	0.66	0.41	0.29	0.15	0.28	0.61	0.098
4-Hydroxybenzaldehyde	0.31	0.79	0.19	1.3	1.7	0.53	0.81	0.12	0.25	0.33	0.41	0.36	1.1	0.24	1.7	0.58	0.46	0.55	0.18	0.29	0.28	0.39
4-Hydoxybenzoic acid	1.6	2.0	3.6	2.0	1.7	0.64	1.2	0.87	2.6	1.6	1.9	1.1	2.8	3.3	1.9	1.7	1.5	1.4	1.4	2.3	2.7	0.86
Acacetin	1.1	0.84	0.42	0.74	6.1	0.66	3.8	0.64	1.5	0.51	0.44	1.1	0.67	0.24	0.65	1.3	0.34	1.9	0.25	0.60	0.43	0.36
Apigenin	0.51	0.40	0.62	0.36	0.66	0.28	1.3	0.18	0.26	0.31	0.26	0.21	0.19	0.59	0.11	0.22	0.15	0.68	0.12	0.15	0.31	0.22
Benzoic acid	0.31	0.89	0.20	1.4	1.6	0.59	0.86	0.13	0.27	0.39	0.35	0.37	0.92	0.23	1.5	0.49	0.48	0.58	0.19	0.28	0.30	0.40
Chrysin	1.5	1.4	0.30	1.0	7.1	0.95	11	1.1	3.0	0.81	1.1	2.0	1.4	0.22	1.3	2.4	0.57	6.2	0.51	1.4	0.85	0.93
Eriodictyol	0.52	0.29	0.30	0.24	0.33	0.19	0.19	0.11	0.13	0.13	0.11	0.25	0.14	0.12	0.10	<LOQ	<LOQ	<LOQ	<LOQ	<LOQ	<LOQ	<LOQ
Ethyl caffeate	0.47	0.37	0.25	0.26	0.25	0.20	0.17	0.16	0.14	0.11	0.10	0.11	0.14	0.10	0.080	0.095	0.088	<LOQ	0.079	<LOQ	<LOD	<LOD
Ethyl gallate	1.7	1.5	1.1	0.85	0.82	0.75	0.66	0.56	0.61	0.46	0.49	0.39	0.33	0.37	0.27	0.33	0.34	0.37	0.30	0.26	0.26	0.25
Ferulic acid	0.63	3.9	0.32	0.49	1.3	0.25	0.59	0.97	0.95	<LOD	0.12	1.3	5.5	1.1	0.73	0.25	<LOD	0.10	<LOQ	0.11	<LOD	<LOD
Galangin	1.3	0.97	0.72	0.69	1.3	0.60	2.4	0.64	0.90	0.39	0.43	0.58	0.42	0.22	0.33	0.53	0.31	1.3	0.25	0.41	0.25	0.25
Genistein	0.54	0.42	0.62	0.36	0.68	0.29	1.2	0.19	0.28	0.33	0.28	0.22	0.19	0.57	0.12	0.21	0.17	0.70	0.12	0.14	0.30	0.22
Kaempferide	1.3	1.0	0.84	0.75	0.66	0.58	0.75	0.43	0.42	0.41	0.33	0.26	0.23	0.24	0.22	0.29	0.19	0.34	0.18	0.18	0.24	0.15
Kaempferol	3.7	2.5	2.1	1.8	4.8	2.2	1.7	1.9	1.5	1.6	0.89	1.7	0.92	1.2	0.87	1.1	1.0	0.76	0.92	0.71	0.91	0.54
Luteolin	4.5	1.5	1.9	1.3	1.8	1.0	1.1	0.70	0.59	0.59	0.65	1.4	1.0	0.84	0.40	0.50	0.45	0.41	0.39	0.37	0.39	0.33
Naringenin	0.95	0.31	0.17	0.082	0.33	0.10	0.11	<LOQ	<LOQ	0.075	<LOQ	0.76	0.16	0.15	0.10	0.17	0.091	0.084	<LOQ	<LOQ	0.099	<LOD
p-Coumaric acid	<LOD	1.9	2.1	3.9	16	2.8	9.6	5.8	8.2	0.95	0.63	1.7	6.1	7.1	7.7	1.5	0.65	1.5	0.49	1.0	0.71	<LOD
Pinobanksin	0.27	0.18	<LOQ	0.14	0.84	0.16	1.7	0.18	0.46	0.20	0.17	0.36	0.26	0.069	0.17	0.39	0.12	1.1	0.10	0.25	0.18	0.15
Pinocembrin	0.63	0.57	0.13	0.42	1.7	0.31	6.4	0.43	1.6	0.25	0.64	0.90	0.68	0.15	0.51	0.92	0.21	3.6	0.18	0.76	0.27	0.56
Quercetin	6.7	2.5	3.5	2.1	10	2.5	1.5	3.7	1.6	2.9	0.78	5.2	2.5	3.3	1.8	2.7	1.4	0.89	1.5	0.83	2.2	0.68
Quinic acid	<LOD	0.45	14	0.53	5.7	0.22	1.1	18	0.20	3.9	1.3	<LOD	0.26	13	0.39	2.6	0.41	1.1	15	0.33	4.4	0.49
Sakuranetin	0.90	1.0	0.19	0.81	15	0.99	8.1	1.3	2.8	0.85	0.69	1.6	1.5	0.39	0.99	4.0	0.66	4.1	0.40	0.97	1.0	0.60
Syringaldehyde	1.3	0.91	0.99	0.60	0.38	0.57	0.47	0.41	0.24	0.36	0.30	0.26	0.20	0.25	0.19	0.30	0.29	0.18	0.24	0.26	0.15	0.16

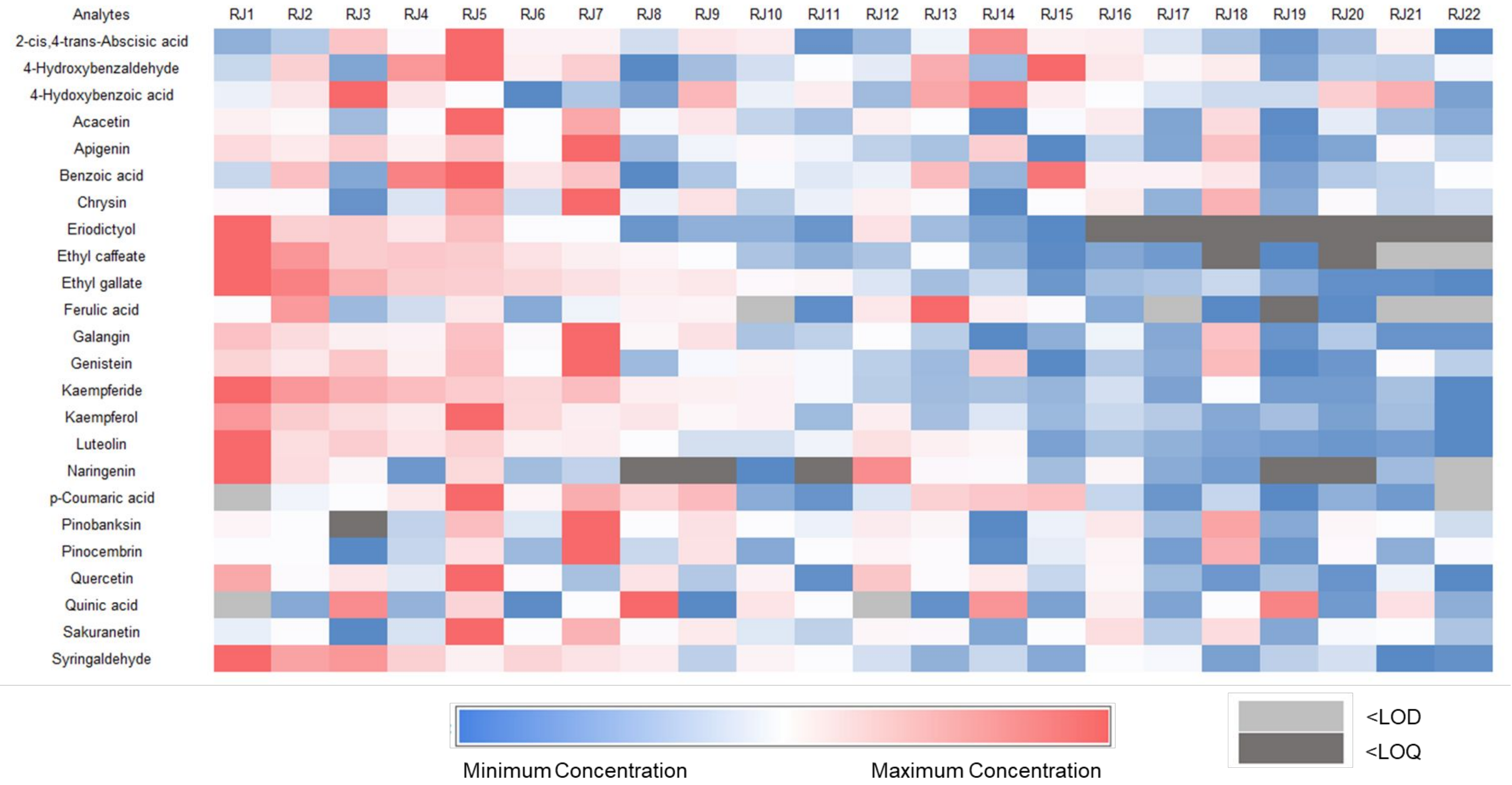

Figure S5: Variation of the concentration of each analyte among the different RJ samples derived from the quantification results.

Table S11: Health benefits of bioactive compounds found in RJ samples.

	Antimicrobial	Anticancer	Anti-inflammatory	Antifungal	Antioxidant	Antibacterial	Antiallergic	Antidiabetic	Neuroprotective	Reference
2-cis,4-trans-Abscisic acid			✓					✓		1
4-Hydroxybenzaldehyde			✓		✓					2,3
4-hydroxybenzoic acid				✓	✓	✓				4
Acacetin		✓					✓			5
Apigenin		✓	✓			✓				5,6
Benzoic acid	✓			✓		✓				7
Chrysin		✓	✓		✓	✓		✓		5,6
Eriodictyol		✓	✓		✓			✓	✓	8
Ethyl caffeate		✓	✓							9
Ethyl gallate		✓			✓	✓				10,11
Ferulic acid			✓		✓				✓	12
Galangin		✓					✓			12
Genistein			✓							12
Kaempferide		✓	✓						✓	12,13
Kaempferol		✓								12
Luteolin		✓	✓		✓					5,6
Naringenin		✓	✓		✓			✓		14
p-Coumaric acid		✓	✓						✓	12
Pinobanksin			✓							12
Pinocembrin	✓	✓	✓		✓				✓	12,15
Quercetin	✓	✓	✓	✓	✓	✓	✓	✓		16
Quinic acid	✓	✓			✓			✓		17
Sakuranetin			✓	✓	✓				✓	18
Syringaldehyde	✓	✓	✓		✓			✓		19

REFERENCES

(1) Magnone, M.; Sturla, L.; Guida, L.; Spinelli, S.; Begani, G.; Bruzzone, S.; Fresia, C.; Zocchi, E. Abscisic Acid: A Conserved Hormone in Plants and Humans and a Promising Aid to Combat Prediabetes and the Metabolic Syndrome. *Nutrients* **2020**,*12* (6) 1-13.

(2) Cheng, J.; Ye, K.; Fu, C.; Zhou, Y.; Chen, Y.; Ma, G.; Chen, S.; Tu, J.; Xiao, H. Comprehensive Assessment of Rice Bran Dietary Fiber on Gut Microbiota Composition and Metabolism during in Vitro Fermentation. *Food Research International* **2024**, *197*, 115231.

(3) Lim, E.-J.; Kang, H.-J.; Jung, H.-J.; Kim, K.; Lim, C.-J.; Park, E.-H. *Anti-Inflammatory, Anti-Angiogenic and Anti-Nociceptive Activities of 4-Hydroxybenzaldehyde*; *Biomolecules and Therapeutics* **2008**, *16* (3), 231-236.

(4) Velika, B.; Kron, I. Antioxidant Properties of Benzoic Acid Derivatives against Superoxide Radical. *Free Radicals and Antioxidants* **2012**, *2* (4), 62-67.

(5) Martinello, M.; Mutinelli, F.; Zooprofilattico, I.; Delle Venezie, S. Antioxidants Antioxidant Activity in Bee Products: A Review. *Antioxidants* **2021**, *10* (1), 71-108.

(6) Özsel Özcan, F.; Aldemĭr, O.; Karabulut, B. *Flavones (Apigenin, Luteolin, Crhysin) and Their Importance for Health*. *FÖ* **2020**, *20* (1), 16–27.

(7) Del Olmo, A.; Calzada, J.; Nuñez, M. Benzoic Acid and Its Derivatives as Naturally Occurring Compounds in Foods and as Additives: Uses, Exposure, and Controversy. *Crit Rev Food Sci Nutr* **2017**, *57* (14), 3084-3103.

(8) Islam, A.; Islam, M. S.; Rahman, M. K.; Uddin, M. N.; Akanda, M. R. The Pharmacological and Biological Roles of Eriodictyol. *Arch. Pharm. Res.* **2020**, *43* (6), 582-592.

(9) Kalló, G.; Kunkli, B.; Győri, Z.; Szilvássy, Z.; Csősz, É.; Tőzsér, J. Compounds with Antiviral, Anti-Inflammatory and Anticancer Activity Identified in Wine from Hungary's

Tokaj Region via High Resolution Mass Spectrometry and Bioinformatics Analyses. *Int J Mol Sci* **2020**, *21* (24), 1-20.

(10) Liu, F.; Zu, X.; Xie, X.; Liu, K.; Chen, H.; Wang, T.; Liu, F.; Bode, A. M.; Zheng, Y.; Dong, Z.; Kim, D. J. Ethyl Gallate as a Novel ERK1/2 Inhibitor Suppresses Patient-Derived Esophageal Tumor Growth. *Mol Carcinog* **2019**, *58* (4), 533-543.

(11) Wang, X.; Li, C.; Yun, F.; Jiang, X.; Yu, L. Preparation and Evaluation of Gallate Ester Derivatives Used as Promising Antioxidant and Antibacterial Inhibitors. *Chem Biodivers* **2021**, *18* (3), 2000913-2000923.

(12) Choudhary, P.; Tushir, S.; Bala, M.; Sharma, S.; Sangha, M. K.; Rani, H.; Yewle, N. R.; Kumar, P.; Singla, D.; Chandran, D.; Kumar, M.; Mekhemar, M. Exploring the Potential of Bee-Derived Antioxidants for Maintaining Oral Hygiene and Dental Health: A Comprehensive Review. *Antioxidants* **2023**, *12* (7), 1452-1483.

(13) Song, B.; Niu, W.; Zhang, S.; Hao, M.; Li, Y.; Chen, Q.; Li, S.; Tong, C. A Mechanistic Review of the Pharmacological Aspects of Kaempferide as a Natural Compound. *Heliyon* **2024**, *10* (19), e38243.

(14) Rao, V. P.; Kiran, S. Flavonoid: A Review on Naringenin. *J. Pharmacogn. Phytochem.* **2017**, *6* (5), 2778–2783.

(15) Elbatreek, M. H.; Mahdi, I.; Ouchari, W.; Mahmoud, M. F.; Sobeh, M. Current Advances on the Therapeutic Potential of Pinocembrin: An Updated Review. *Biomedicine and Pharmacotherapy* **2023**,114032-114045.

(16) Aghababaei, F.; Hadidi, M. Recent Advances in Potential Health Benefits of Quercetin. *Pharmaceuticals* **2023**, *16* (7), 1020-1051.

(17) Benali, T.; Bakrim, S.; Ghchime, R.; Benkhaira, N.; El Omari, N.; Balahbib, A.; Taha, D.; Zengin, G.; Hasan, M. M.; Bibi, S.; Bouyahya, A. Pharmacological Insights into the Multifaceted Biological Properties of Quinic Acid. *Biotechnol Genet Eng Rev* **2022**, 1-30.

(18) Junaid, M.; Basak, B.; Akter, Y.; Afrose, S. S.; Nahrin, A.; Emran, R.; Shahinozzaman, M.; Tawata, S. Sakuranetin and Its Therapeutic Potentials-a Comprehensive Review. *Journal of Biosciences- Section C* **2023**, 27-48.

(19) Wu, J.; Fu, Y. S.; Lin, K.; Huang, X.; Chen, Y. jing; Lai, D.; Kang, N.; Huang, L.; Weng, C. F. A Narrative Review: The Pharmaceutical Evolution of Phenolic Syringaldehyde. *Biomedicine and Pharmacotherapy* **2022**, *153*, 113339-113352.
